# Comparison of Morphological and DNA‐Based Identification Methods to Assess Earthworm (Clitellata: Lumbricidae) Diversity at 25 Permanent Soil Monitoring Sites in Germany

**DOI:** 10.1002/ece3.71155

**Published:** 2025-03-30

**Authors:** Stephan Jänsch, Daniela Alves, Luís Cunha, Paul Henning Krogh, Tiago Natal‐da‐Luz, Verónica Rojo, Jörg Römbke, Rumakanta Sapkota, Adam Scheffczyk, Rüdiger M. Schmelz, Letícia Scopel, José Paulo Sousa, Joaquín Vierna, Antón Vizcaíno

**Affiliations:** ^1^ ECT Oekotoxikologie GmbH Flörsheim am Main Germany; ^2^ CloverStrategy Lda. Coimbra Portugal; ^3^ Centre for Functional Ecology, Associate Laboratory TERRA, Department of Life Sciences University of Coimbra Coimbra Portugal; ^4^ Department of Ecoscience Aarhus University Aarhus Denmark; ^5^ AllGenetics & Biology SL Oleiros (A Coruña) Spain; ^6^ Department of Environmental Science Aarhus University Roskilde Denmark; ^7^ Freelance Researcher A Coruña Spain

**Keywords:** 16S, COI, community DNA, environmental DNA, metabarcoding

## Abstract

The most important reason for the lack of a German nationwide and standardised survey of soil organisms is probably the time‐consuming and expensive identification of soil invertebrates. The present contribution should contribute to solving this problem. Earthworms and soil were sampled at 25 sites, the animals were identified morphologically and by community DNA (comDNA) and environmental DNA (eDNA) metabarcoding. The comparison of results showed that comDNA detected more species (3.6 on average) than eDNA (3.0) and morphological identification (2.8). In contrast, eDNA, on average, detected a similar number of species as morphological identification. However, some species appear to have a different probability of being detected by eDNA than others, depending on their abundance, behaviour, biology or body size. All three identification methods can differentiate between sites with different species composition, and the degree of separation can vary depending on the identification method. The relative proportion of eDNA reads shows potential as a surrogate of relative abundance/biomass for endogeic but not for anecic species. The overall aim of the ‘MetaSOL’ project (which the present contribution originated from) was to develop recommendations for efficient and routinely implementable monitoring of soil fauna. The results showed that genetic identification methods are suitable for earthworms. Before genetic identification methods can be introduced into official practice, key preconditions such as comprehensive, well‐curated and quality‐controlled DNA reference databases and method standardisation must be addressed. Robust indices of soil health based on soil organism data need to be developed. The inclusion of further groups in addition to earthworms should be examined.

## Graphical abstract

The present contribution should contribute to solving the problem of the time‐consuming and expensive identification of soil invertebrates in the standardised survey of soil organisms. Earthworms and soil were sampled at 25 sites; the animals were identified morphologically and by community DNA (comDNA) and environmental DNA (eDNA) metabarcoding, and the results compared. The results showed that genetic identification methods for earthworms are generally suitable to be included in an efficient and routinely implementable monitoring of soil fauna, but that several preconditions must first be met.

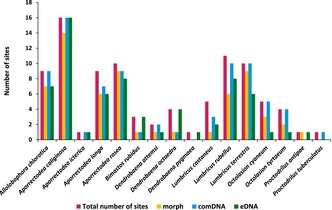

## 1 Introduction

The monitoring and assessment of the diversity of soil invertebrates has made great progress in recent years at the European level (Creamer et al. 2016; Griffiths et al. 2016) and in Germany (e.g., Jänsch et al. 2013; Toschki et al. 2021). However, Germany lacks a standardised monitoring of soil organisms. A comprehensive infrastructure is available in the form of around 800 well‐characterised permanent soil monitoring sites (BDF) (Toschki et al. 2015), but not all federal states carry out soil biological investigations on their BDFs. The most important reason for this is probably the time‐consuming and, due to the lack of trained taxonomists, expensive identification of soil invertebrates. The work described in this contribution was part of a broader project supported by the German Environment Agency (‘MetaSOL’) that intended to help solve this problem by recording important soil organism groups at selected sites (primarily BDF) and then identifying these animals using both ‘classical’ morphological and ‘modern’ genetic methods (metabarcoding). The ‘MetaSOL’ project's overall aim was to develop recommendations for efficient and routinely implementable monitoring of selected soil organism groups (earthworms, collembolans and enchytraeids) within the framework of the German BDF programme. This contribution focuses on the results of the method comparison for earthworms, more specifically Lumbricidae, the only terrestrial earthworm family occurring in Germany (Lehmitz et al. 2014). Earthworms play a crucial role in maintaining soil health and providing ecosystem services (FAO et al. 2020). They improve soil structure by aerating and mixing it, which enhances water infiltration and nutrient distribution. Their burrowing activities also help break down organic matter, releasing nutrients for plants. Additionally, earthworms increase microbial activity in the soil, contributing to nutrient cycling and decomposition (Edwards and Arancon 2022).

An important prerequisite for the protection of soil health is sufficient knowledge of the occurrence (diversity, abundance) of soil organisms at a specific site or in the respective soil. Therefore, robust data must be collected using standardised methods in comprehensive and representative monitoring studies (Gardi et al. 2009; Faber et al. 2013; Kotschik et al. 2023). An important reason for the neglected recording of soil biodiversity so far may be the enormous number of organisms and species in the soil (Jeffrey et al. 2010). Indeed, it is difficult to record this high biodiversity using ‘classical’ morphological methods, partly because of the large amount of time (and thus high costs) required to identify many individuals, and partly because of the increasing shortage of well‐trained taxonomists. This problem could at least be minimised by using ‘modern’ genetic methods (e.g., Porco et al. 2013). In soil biology, these new methods have so far mainly been used with earthworms (Bienert et al. 2012; Pérez‐Losada et al. 2012; Decaens et al. 2013; Cuartero et al. 2025). The standardisation of practical steps, from sampling to the actual laboratory work to the general conditions (e.g., storage of samples), is also increasing considerably (e.g., Straube and Juen 2013; ISO 2017). However, the question of the extent to which genetic methods can be used to determine not only the occurrence of a species in a soil sample but also the respective number of organisms of a species has not yet been resolved.

For these reasons, there is an urgent need to further develop the genetic methods primarily used to date such as deoxyribonucleic acid (DNA) barcoding. DNA barcoding uses fragments of a ‘standard gene’ for the molecular identification of species; in the case of invertebrates, this is usually cytochrome oxidase subunit I (COI). There are two options for further development: using additional genes (primarily from the cell nucleus) and developing DNA metabarcoding (Taberlet, Coissac, Pompanon, et al. 2012) combining DNA barcoding with high throughput sequencing to detect the taxonomic composition of the DNA present in a complex sample. In this context, a complex sample is made up of a mix of organisms or their DNA. In this paper, we will distinguish two types of samples: ‘community DNA’ or ‘comDNA’, which refers to the DNA isolated from a sample containing tissue of a mixture of organisms extracted from the soil; and ‘environmental DNA’ or ‘eDNA’, which refers to the DNA isolated from whole soil samples that may contain tissue remnants, individual cells, or free DNA of a wide variety of species of meso‐ and macrofauna as well as microorganisms (Taberlet, Coissac, Hajibabaei, et al. 2012; Thomsen and Willerslev 2015; Zinger and Philippe 2016; Taberlet et al. 2018).

## 2 Material and Methods

### 2.1 Study Sites

**FIGURE 1:**
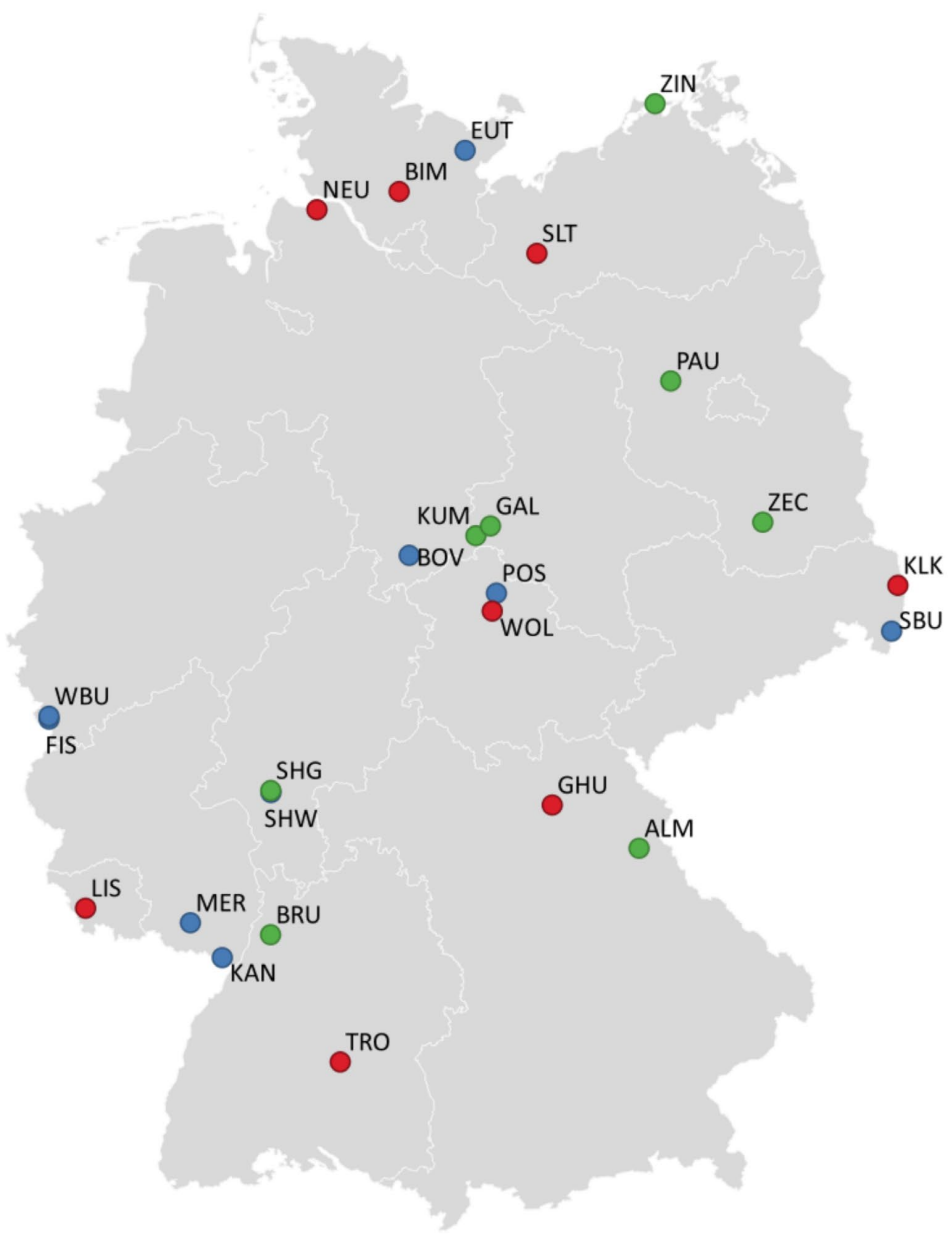
Map representing the location of the 25 selected sites in Germany. Each coloured circle indicates location and land use (red = arable land, green = grassland, blue = forest; for site abbreviations see Table 1).

The practical work was based on the sampling of 25 sites in Germany. The main basis for the selection of the sampling sites was the 800 BDFs in the German permanent soil monitoring programme (Toschki et al. 2015). BDFs for which relevant information was already available were of particular interest. It was aimed to ensure a balance between the three main land use classes (arable land, grassland, forest) and in the geographical distribution of the sampling sites (Figure 1). Well‐characterised sites without BDF status were also included in the selection (e.g., areas of the Bavarian State Institute for Agriculture and the Eifel National Park, sampled as part of the ‘Edaphobase II’ project; Toschki et al. 2021; Table 1). While the selected sites are not fully representative of Germany or its entire earthworm species diversity, efforts were made to include a wide range of habitats and soil properties as a solid basis for comparing earthworm identification methods.

**TABLE 1:** List of the 25 selected sites with abbreviation, status (BDF = permanent soil monitoring site; EB = “Edaphobase” project), names, federal state (FS), land use (LU: A = arable land, G = grassland, W = forest), location, date of sampling, coordinates, soil pH (CaCl_2_), C/N ratio, organic carbon [%], total nitrogen [%] and soil texture.

Code	Status	State‐/EB‐specific name	FS	LU	Site	Sampling date (dd.mm.yyyy)	Coordinates (WGS 84)	pH	C/N	C_org_	*N*_tot_	Texture
ALM	EB	Almesbach Stallkoppel EB_BY11	BY	G	Weiden i.d. Oberpfalz	26.05.2021	49.681233; 12.200329	6.4	10.1	6.51	0.65	Su3
BIM	BDF	BDF 26: Bad Bramsted	SH	A	Bimöhlen	04.11.2019	53.923511; 9.917934	5.3	15.2	3.4	0.23	Ss
BOV	BDF	BF80HABU	NI	W	Bovenden	18.05.2021	51.595822; 9.974223	4.2	14.0	29.7	2.12	Ut4
BRU	BDF	Bruchsal	BW	G	Forst	22.10.2019	49.171535; 8.573805	6.7	16.2	2.92	1.6	lS
EUT	BDF	BDF 20: Wüstenfelde	SH	W	Eutin	05.11.2019	54.179276; 10.641967	4.4	15.2	7.06	0.47	Sl4‐Ls3
FIS	EB	Fichte Süd EB_NW12	NW	W	Monschau	10.10.2019	50.52131; 6.34056	3.0	20.0	17.0	0.83	Uls
GAL	EB	Galgenberg EB_ST31	ST	G	Elbingerode	16.10.2019	51.773793; 10.814907	6.1	12.3	10.1	0.83	Us
GHU	EB	Großenhül In der Huh EB_BY33	BY	A	Thurnau	26.10.2018	49.978414; 11.358906	5.8	9.2	1.72	0.19	Ut2
KAN	BDF	BDF 1: Kandel/Schaidt	RP	W	Kandel	29.10.2019	49.022290; 8.105280	3.4	14.6	7.47	0.51	Su2
KLK	EB	Klein‐Krauscha EB_SN34	SN	A	Neißeaue	20.10.2021	51.25657; 14.94617	4.2	11.98	2.29	0.19	Su2
KUM	EB	Kümmelwiese EB_ST21	ST	G	Braunlage	30.10.2018	51.713757; 10.662652	4.7	11.7	9.10	0.78	Ut2
LIS	BDF	BDF 6606–0186: Saarlouis‐Lisdorf	SL	A	Lisdorf	09.10.2019	49.320666; 6.763109	6.7	19.2	3.64	0.19	Ut3
MER	BDF	BDF 2: Merzalben	RP	W	Merzalben	29.10.2019	49.242356; 7.790209	3.5	17.0	6.79	0.40	Sl3
NEU	BDF	BDF014‐L Neuhäuserfelde	NI	A	Neuhaus	04.11.2019	53.810873; 9.028747	5.6	10.2	0.86	0.09	Uls
PAU	BDF	BDF 17: Paulinenaue	BB	G	Paulinenaue	06.11.2019	52.655927; 12.746113	6.3	12.3	34.2	2.78	n.a.
POS	BDF	BDF 19 Possen (POSS)	TH	W	Sondershausen	31.10.2018	51.343192; 10.858749	4.1	17.3	6.9	0.4	Ut4
SBU	EB	Schlegeler Buchberg EB_SN22	SN	W	Zittau‐Schlegel	20.10.2021	50.96841; 14.84632	4.9	13.90	14.7	1.05	Su4
SHG	BDF	BDF 53: F‐SH2 (Schwanheim II)	HE	G	Kelsterbach	18.10.2019	50.094220; 8.567582	6.6	9.3	3.22	0.34	Ls3
SHW	BDF	BDF 52: F‐SH1 (Schwanheim 1)	HE	W	Kelsterbach	18.10.2019	50.081072; 8.569127	3.5	10.7	6.66	0.61	Lu
SLT	BDF	BDF‐L Nr. 24 Sülstorf	MV	A	Sülstorf	03.11.2021	53.507608; 11.385792	5.4	12.5	1.10	0.09	Ss
TRO	BDF	Trochtelfingen	BW	A	Trochtelfingen	23.10.2019	48.357624; 9.249132	5.8	n.a.	n.a.	n.a.	Tl
WBU	EB	Wächterbuche EB_NW21	NW	W	Monschau	10.10.2019	50.54022; 6.33821	3.4	17.4	15.1	0.86	Uls
WOL	BDF	BDF 13 Wolferschwenda (WSWE)	TH	A	Wolferschwenda	15.10.2019	51.229550; 10.811587	6.1	8.0	1.6	0.2	Ut4
ZEC	BDF	BDF 28: Zeckerin	BB	G	Zeckerin	21.10.2021	51.721298; 13.615597	6.3	10.4	6.5	0.63	Sl3
ZIN	BDF	BDF‐L Nr. 5 Zingst	MV	G	Zingst	03.11.2021	54.430655; 12.734537	4.9	12.2	1.64	0.13	Ss

*Note:* Data sources of the soil data (pH, C/N, C_org_, N_tot_, texture): Status BIM = Woloszczyk et al. (2018); BOV = Nordwestdeutsche Forstliche Versuchsanstalt; BRU, TRO = Landesanstalt für Umwelt Baden‐Württemberg; EB = Toschki et al. (2021); EUT = Woloszczyk et al. (2019); KAN, MER = Forschungsanstalt für Waldökologie und Forstwirtschaft Rheinland‐Pfalz; LIS = Landesamt für Umwelt‐ und Arbeitsschutz Saarland; n.a. = not available; NEU = Landesamt für Bergbau, Energie und Geologie Niedersachsen; PAU, ZEC = Landesamt für Umwelt Brandenburg; POS, WOL = Landesamt für Umwelt, Bergbau und Naturschutz Thüringen; SHG, SHW = Hessisches Landesamt für Naturschutz, Umwelt und Geologie; SLT, ZIN = Landesamt für Umwelt, Naturschutz und Geologie Mecklenburg‐Vorpommern. Federal states: BB = Brandenburg; BW = Baden‐Württemberg; BY = Bavaria; HE = Hesse; Ls3 = medium sandy loam; Lu = silty loam; MV = Mecklenburg Western Pomerania; NI = Lower Saxony; NW = Northrhine‐Westphalia; RP = Rhineland‐Palatinate; SH = Schleswig‐Holstein; SL = Saarland; Sl3 = medium loamy sand; Sl4 = strongly loamy sand; SN = Saxony; Soil texture: lS = loamy sand; Ss = pure sand; ST = Saxony‐Anhalt; Su2 = slightly silty sand; Su3 = medium silty sand; Su4 = strongly silty sand; TH = Thuringia; Tl = loamy clay; Uls = sandy‐loamy silt; Us = sandy silt; Ut2 = slightly clayey silt; Ut3 = medium clayey silt; Ut4 = strongly clayey silt.s.

### 2.2 Sampling and Morphological Identification

**FIGURE 2:**
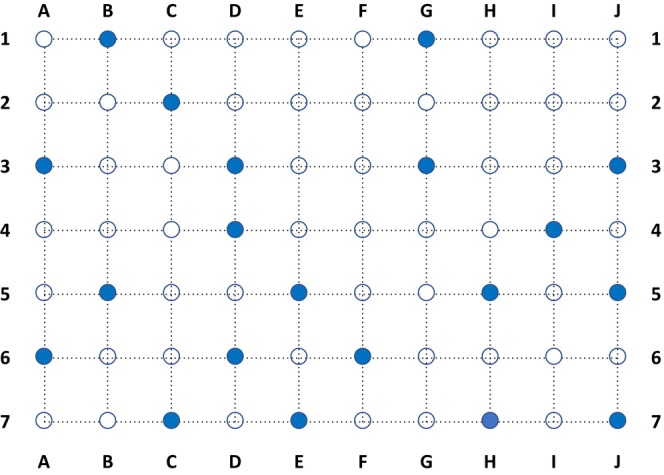
Sampling layout for lumbricid eDNA‐samples (ø = 5 cm, depth: 0–20 cm); spacing of sampling points: 1 m. Blue dots: Soil cores for lumbricid eDNA‐samples, white dots: Soil cores taken to investigate Collembola and Enchytraeidae.

Lumbricids were sampled following ISO standard 23611‐1 (ISO 2018). In accordance with the standard, the sites were sampled in spring and autumn (i.e., during periods of highest earthworm activity to minimise the seasonal influence on abundance and detectability) of 2018 to 2021 (Table 1), with no samplings in 2020 due to the COVID‐19 pandemic. The topsoil (including litter layer, if present) was excavated with a spade to a depth of 20 cm at five points per site around the area for sampling soil cores for eDNA isolation over an area of 50 cm x 50 cm (= ¼ square metre). The excavated soil samples were hand‐sorted and searched for lumbricids directly at the sampling site. Five litres of a 0.01%‐allyl isothiocyanate (AITC) solution were poured into the excavated sampling hole to extract deep‐burrowing earthworms. Earthworms were collected in plastic containers filled with 70%‐ethanol to fix and preserve the tissue. In addition, 20 soil samples (Ø 5 cm, 0–20 cm) were taken for eDNA extraction at the same sites. Sampling was performed using probes equipped with sleeves that were exchanged for each eDNA composite sample to avoid cross‐contamination between sites. The soil cores were taken at one‐metre intervals using a pre‐determined scheme over an area of 6 × 9 m to ensure each area was as representative as possible (Figure 2).

In the laboratory, the earthworms were transferred to fresh 70%‐ethanol. All adult and subadult specimens were identified under a low magnification stereo microscope to species level, and most juveniles were identified at the genus level. Pieces of individuals were identified to species level if the required morphological features were retained, remaining pieces were recorded as ‘not determinable’. The total wet weight of all individuals (stored in70%‐ethanol) per taxon was measured. At the same time, the affiliation to one of the three ecological groups (epigeic, endogeic, anecic) was recorded (Bouché 1977). The morphological identification of the earthworms was based on the key of Sims and Gerard (1999), whereby in cases of doubt Graff (1953), Bouché (1972) or Csuzdi and Zicsi (2003) were consulted.

### 2.3 Preparation of comDNA Samples

After morphological identification and weighing, a tissue fragment (1–3 mm) from the posterior part of the body (irrelevant for morphological identification) was removed from each specimen (including juveniles and fragments), avoiding the digestive tract if possible. The collected tissue fragments were pooled in a 2‐mL sample tube and filled with 96%‐ethanol to a volume ratio of 3:1 (v:v) ethanol: tissue. Samples containing many tissue fragments were divided into several sample tubes. The sample vials were then stored in the refrigerator until further processing.

### 2.4 eDNA Extraction From Soil Samples

DNA was extracted, as previously described in Taberlet, Prud'Homme, Campione, et al. (2012); Taberlet et al. (2018) and Pansu et al. (2015), allowing processing of large quantities of soil (several kg). The 20 individual soil cores per site were pooled into one composite sample. In the first step, the respective sample was extracted using a 0.12 M phosphate‐buffered saline solution (PBS; Na_2_HPO_4_: NaH_2_PO_4_ = 5.3: 94.7) adjusted to pH ≥ 8.0. The materials used, such as the wide‐neck bottle, funnel, spoon, glass rod, scissors, pipette tips, and the phosphate buffer solution, were autoclaved before use. The work surface, pipettes, and the rack for the prepared pipette tips were cleaned with bleach (0.05% NaClO) and 70%‐ethanol. In addition, the work surface and materials were exposed to ultraviolet light for approx. 15 min. An amount of phosphate buffer solution corresponding to the weight of the soil sample was added; for example, 1700 g soil (wet weight) was mixed with 1700 mL buffer solution. The mixture was stirred with a glass rod and agitated at 400 rpm for 15 min. A volume of approx. 200 mL of the phosphate buffer without soil as negative control (NC) was also shaken for 15 min. The NC was prepared for each batch of phosphate buffer. The pipettes used were wrapped with parafilm to avoid contamination with DNA. Immediately after shaking (the solution should not settle), 1.8 mL of the supernatant was pipetted 10 times per sample and 1.8 mL of the NC twice per working day into 2‐mL Eppendorf tubes. The tubes were stored at ≤ −18°C. Four of the 10 replicates were sent for further processing on dry ice, and six retention samples remained stored. The samples were thawed in the Eppendorf tubes and centrifuged at 1000 G for 10 min. In total, 700 μL of the supernatant were transferred to new Eppendorf tubes, and the DNA was extracted using the ‘NucleoSpin Soil’ kit (Macherey‐Nagel).

### 2.5 DNA Extraction From comDNA Samples

The bulk tissue samples were pooled per sampling site for comDNA analyses. For each sampling site, a 1‐mm^2^ piece was taken from each individual fragment and pooled into as few tubes as possible for DNA extraction.

DNA extraction was performed with the E.Z.N.A Tissue DNA kit (Omega Bio‐tek) using the manufacturer protocol, including overnight Lysis at 55°C after the addition of Proteinase K. The DNA was resuspended in a final volume of 100 μL of elution buffer. An extraction negative control was included in every DNA extraction round and treated as a regular sample to check for cross‐contamination.

### 2.6 DNA Metabarcoding Library Preparation and Sequencing

DNA metabarcoding libraries were prepared using two different primer sets: comDNA libraries were constructed with a modified version of the ‘Leray’ primer pair (Leray et al. 2013), using the forward primer ‘MetaSOL mlCOIintF’ (5′ GGW ACW GGD TGA ACW GTW TAY CCH CC 3′) and the reverse primer ‘MetaSOL HCO2198’ (5′ TAW ACT TCW GGG TGW CCR AAR AAY CA 3′), which target a 313‐base pair (bp) fragment of the mitochondrial COI gene. The modifications were designed to increase primer degeneracy, thereby improving amplification efficiency for Lumbricidae. For eDNA, amplicon libraries were constructed using the primer pair ‘ewD’ (5′ ATT CGG TTG GGG CGA CC 3′) and ‘ewE’ (5′ CTG TTA TCC CTA AGG TAG CTT 3′) (Bienert et al. 2012), which amplifies a fragment of about 70 bp of the mitochondrial 16S ribosomal gene. This choice of different primers for comDNA and eDNA analysis, respectively, was based on results of a preceding benchmarking study on the suitability of these two metabarcoding markers for eDNA species detection, comparing them with results of morphological methods. The shorter of the 16S marker, compared to that of the COI marker, may improve PCR amplification of extracellular DNA in environmental samples, which is expected to be highly degraded (Bienert et al. 2012; Pansu et al. 2015). Besides, the 16S primers target highly conserved regions across earthworm species decreasing PCR biases and ensuring the detection of all the target species. Conversely, since the 16S gene is less variable than the COI, it offers a lower taxonomic resolution and hence may fail to discriminate closely related species. We found that COI offered better taxonomic resolution in comDNA samples, particularly for detecting potential cryptic species. In contrast, the shorter 16S marker proved more effective for eDNA, detecting more earthworm taxa while retrieving fewer non‐target reads.

Illumina sequencing primer sequences were attached to these primers at their 5' ends. A two‐step PCR protocol was applied for library preparation (see e.g., Vierna et al. 2017). In the first amplification step, PCRs were carried out in triplicate in a final volume of 12.5 μL, containing 2.5 μL of template DNA (diluted 1:10), 0.5 μM of the primers, 6.25 μL of Supreme NZYTaq 2x Green Master Mix (NZYTech), 1X CES (Ralser et al. 2006), and ultrapure water up to 12.5 μL. The cycling conditions for the reaction mixture were: an initial denaturation step at 95°C for 5 min, followed by 35 cycles of denaturing at 95°C for 30 s, annealing at 53°C (COI) or 48°C (16S) for 45 s, 72°C for 45 s, and a final extension step at 72°C for 7 min.

Before the second amplification, the PCR triplicates of each sample were pooled together, purified with Mag‐Bind RXNPure Plus magnetic beads (Omega Biotek) and used as a template for the second PCR that was performed to add the oligonucleotide indices required for multiplexing different libraries in the same sequencing pool. The PCR conditions were identical to those described previously, except that only five thermal cycles were used at 60°C as the annealing temperature.

A negative control that contained no DNA was included in every PCR round to check for contamination during library preparation. Resulting PCR amplicon products were run on 2%‐agarose gels stained with GreenSafe (NZYTech) and imaged under UV light to verify the library size. Finished libraries were purified using the Mag‐Bind RXNPure Plus magnetic beads and equimolarly pooled using DNA concentration amounts measured by the Qubit dsDNA HS Assay (Thermo Fisher Scientific). The libraries were sequenced in a fraction (1/2) of a MiSeq PE300 flow cell (Illumina Inc. San Diego, CA, USA).

### 2.7 Quality Control, Processing of Sequencing Data and Inference of Amplicon Sequence Variants (ASVs)

Non‐biological sequences (indexes, and sequencing adapters) observed at the ends of some reads were trimmed off using Cutadapt v3.5 (Martin 2011). Then, the obtained COI and 16S amplicon reads were separately processed using QIIME 2 (release 2021.4) (Bolyen et al. 2019). Specifically, we used the tool DADA2 (Callahan et al. 2016), implemented in QIIME2, to remove the PCR primers, filter the reads according to their quality, denoise and infer ASVs, merge the forward and reverse reads, and remove chimaeric sequences.

The first step in the DADA2 pipeline consisted of trimming and filtering the data to avoid low‐quality scores. In this case, after checking the read quality profiles, the 16S reads were truncated at position 60 for both the forward and reverse reads. In the case of the COI data set, forward reads were truncated at position 239 and reverse reads at position 187. All subsequent steps in the DADA2 pipeline were run with default settings. Following truncation, error rates were learned from the dataset to denoise, using the parametric error model implemented in DADA2. Before the inference of sequence variants, dereplication of the dataset was carried out, that is, the combination of all identical reads into unique reads to reduce computational effort. Then, these dereplicated forward and reverse reads were used to infer ASVs with the core sample inference algorithm (Callahan et al. 2016). Consecutively, corresponding R1 and R2 reads were merged into pairs by overlapping a minimum of 12 identical base pairs. The DADA2 pipeline included a final step to reduce the impact of artefacts in the dataset. These artefacts, such as chimaeras, are produced during PCR and sequencing and could lead to an overestimation of the number of merged ASVs if not removed. The resulting output of the DADA2 pipeline was a table containing the count of occurrences of every observed ASV in each sample. This table could be readily used for taxonomic assignment.

### 2.8 Taxonomic Assignment

Taxonomic assignment of each ASV was conducted using the *feature‐classifier classify‐consensus‐vsearch* algorithm (Rognes et al. 2016) implemented in QIIME 2 against MIDORI Reference 2, based on GenBank release 249 (April 2022), for the 16S data set and MetaCOXI (Balech et al. 2022) for the COI data set. Considering the high COI and 16S interspecific variability reported in earthworms, alongside the presence of highly divergent genetic lineages likely representing cryptic species (e.g., Decaens et al. 2013; Klarica et al. 2012; Porco et al. 2018; Rougerie et al. 2009), we ran this algorithm using three different identity thresholds: 97%, 95%, and 88%. For the 88%‐threshold, we enabled the ‘top‐hits‐only’ option to retrieve only the sequences with the highest percentage of identity between the query and reference sets. We then extracted and compared the taxonomic information associated with the ASVs assigned to earthworm taxa by the different thresholds applied. Any discrepancies were resolved by manually comparing the ASVs sequences against the MIDORI2 and MetaCOXI databases, using the BLAST+ tool blastn (v2.12.0, Camacho et al. 2009). As a norm, we accepted the taxonomy of the best‐matching reference sequence (i.e., the one presenting the highest percentage of identity with the ASV sequence) and applied a 95%‐identity threshold for species‐level identifications. When an ASV sequence shared equal identity values above the 95%‐threshold with reference sequences belonging to different species, it was classified to the lowest shared taxonomic level (e.g., genus).

The resulting ASV tables were further filtered using the *feature‐table filter‐features* function in QIIME 2 to remove singletons (i.e., ASVs containing only one member sequence in the whole dataset), as well as ASVs occurring at a frequency below 0.01% in each sample, which could potentially be attributed to mistagging (Bartram et al. 2016; Esling et al. 2015; Guardiola et al. 2016; Illumina 2017). In addition, we eliminated any ASVs found in the negative control samples with a read count equal to or higher than the lowest count observed in a test sample. Finally, the ASVs that remained unidentified or were assigned to non‐target taxa were removed from the filtered ASV table before performing the downstream analyses.

### 2.9 Adjustment of Species Lists for Method Comparison

To be able to meaningfully compare the species lists and quantities (abundance, biomass, number of sequencing reads) per site obtained with the three different identification methods, various adjustment steps were carried out. Firstly, any discrepancies between the morphological and DNA metabarcoding identifications were resolved by manually comparing the ASV sequences against the curated reference databases used for taxonomic assignment (i.e., MIDORI and MetaCOXI) and the non‐curated NCBI Nucleotide database (https://www.ncbi.nlm.nih.gov/nucleotide/), in addition to cross‐referencing with available literature. These discrepancies primarily concerned taxa with known cryptic diversity, such as the genus *Aporrectodea* Örley, 1885 (specific examples for this genus are provided in Appendix S1).

Finally, to assess whether a species recognised with one method was also found with another method or not, the species lists generated with the three different methods were trimmed to include only those taxa that are characterised at the morphological and the DNA level. This meant, for example, the elimination of ASVs assigned at the genus level (e.g., “*Aporrectodea* sp.”), and the merging of cryptic lineages within a morphologically defined species into one species.

### 2.10 Statistical Analyses

The adjusted species lists obtained from the comDNA, eDNA, and morphological identifications were converted to presence/absence data. UpSet plots comparing the number of species detected by each method in each sampling site were generated with the Intervene tool (Khan and Mathelier 2017).

Species richness estimates were also represented in a scatter plot using the ggplot2 package in R (R‐Team‐Core 2019; Wickham 2016). Using presence/absence data, beta diversity between the communities was estimated using the Jaccard index via the *qiime diversity beta* plugin of QIIME 2. The Jaccard distances were visualised using a Principal Coordinates Analysis (PCoA) plot with the *qiime diversity pcoa* function.

The metabarcoding results do not provide any direct data on the abundance or biomass of the species detected in this way, but only an initially uninformative number of sequencing reads per ASV. For this reason, we investigated the association between eDNA metabarcoding reads and species abundance (number of individuals) or species biomass (for absolute numbers, see Appendix S2) by correlation test using the cor.test()function in R (R‐Team‐Core 2019). To do so, the ASV abundance tables were first transformed to relative abundance data using the decostand function (method = ‘total’) from the R package vegan v.2.5–7 (Oksanen et al. 2020). As the relative abundance data were not normally distributed (data not shown), we calculated the non‐parametric Spearman's rank correlation coefficient (*R*, rho) and its associated *p*‐value. Scatter plots showing the association between species abundance and eDNA reads, and between species biomass and eDNA reads were generated using the ggpubr package in R (Kassambara 2020).

The correlation tests and scatter plots were conducted not only for the entire dataset (including all species) but also for each species individually and for species clustered according to their ecological groups: endogeic ( *Allolobophora chlorotica* (Savigny, 1826), *Aporrectodea caliginosa* (Savigny, 1826), *Aporrectodea icterica* (Savigny, 1826), *Aporrectodea rosea* (Savigny, 1826), *Octolasion cyaneum* (Savigny, 1826), *Octolasion tyrtaeum* (Savigny, 1826), *Proctodrilus antipae* (Michaelsen, 1891), and *Proctodrilus tuberculatus* (Černosvitov, 1935)), epigeic (*Bimastos rubidus* (Savigny, 1826), *Dendrobaena attemsi* (Michaelsen, 1902), *Dendrobaena octaedra* (Savigny, 1826), *Dendrobaena pygmaea* (Savigny, 1826), *Lumbricus castaneus* (Savigny, 1826), and *Lumbricus rubellus* Hoffmeister, 1843), or anecic ( *Aporrectodea longa* (Ude, 1885) and *Lumbricus terrestris* Linnaeus, 1758).

## 3 Results

**FIGURE 3:**
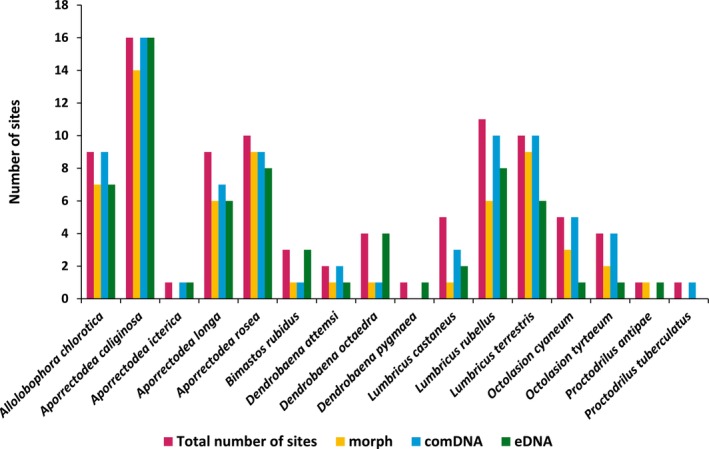
Number of sites where each earthworm species was detected with all identification methods combined or with each individual identification method.

A total of 1754 earthworm specimens and 792 g biomass (fresh weight) were collected. At the 25 sampled sites, 12 different earthworm species were morphologically identified, which corresponds to approx. 26% of the 47 species detected in Germany to date (Lehmitz et al. 2014; Szederjesi et al. 2024). The six species most frequently detected morphologically (in descending order; Figure 3) were *Aporrectodea caliginosa* , *Aporrectodea rosea* , *Lumbricus terrestris* , *Allolobophora chlorotica* , *Aporrectodea longa* , and *Lumbricus rubellus* , followed by *Octolasion cyaneum* , *Octolasion tyrtaeum* , *Bimastos rubidus*, *Dendrobaena attemsi* , *Dendrobaena octaedra* , *Lumbricus castaneus* , and *Proctodrilus antipae*. The DNA‐based methods additionally detected *Aporrectodea icterica* , *Dendrobaena pygmaea* , and *Proctodrilus tuberculatus* (Figure 3). Overall, DNA‐based methods provided good taxonomic resolution, with the majority of Lumbricidae ASVs successfully assigned to the species level (235 out of 245 COI ASVs from comDNA samples and 65 out of 68 16S ASVs from eDNA samples). The remaining ASVs could only be classified at the genus or family levels, either due to a lack of matches above the minimum identity threshold in reference databases or the presence of equally good matches belonging to different species or genera.

**FIGURE 4:**
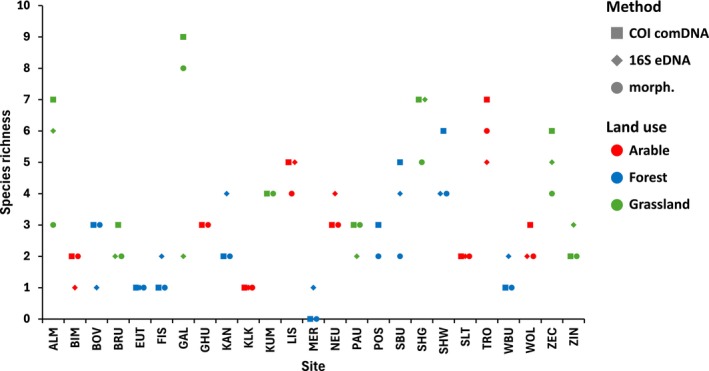
Species richness of earthworms per identification method and location (see Table 1 for location abbreviations). No 16S eDNA data are available for sites GHU, KUM, and POS.

Based on the morphological identification results, an average of 3 species per arable site, 2 species in forests, and 4 species on grassland sites were identified. The average total abundance per site was 70 ind./m^2^ in arable sites, 17 ind./m^2^ in forests, and 87 ind./m^2^ in grassland sites (Appendix S2). The lowest abundance (0–38 ind./m^2^) and number of species (0–4) were thus found at the forest sites. The highest number of species (8) was found in the ‘Galgenberg’ grassland (Figure 4), and the highest abundance (296 ind./m^2^) at the ‘Zeckerin’ grassland site. During the sampling campaign, it was found that the soil at some sampling sites, especially in forests, was very dry, which often led to a low number of earthworm individuals. For example, no earthworm specimens were found at the highly acidic ‘Merzalben’ site, with only one species detected by eDNA (Figure 4). A comparison of the earthworm species identified by the three identification methods per site using up‐set diagrams and species lists can be found in Appendix S3.

### 3.1 Species Richness per Method at All Sites

It was found that comDNA metabarcoding detected more species per site (3.6 on average) than eDNA metabarcoding (3.0) and morphological identification (2.8). On the other hand, a similarly high number of species was detected with eDNA metabarcoding and morphological identification. Figure 4 shows the number of species detected per method at each site. The number of species detected by each method varied significantly; in some cases, the results were very different, while in others, they were quite similar, particularly when the overall number of species detected was low. comDNA metabarcoding detected the highest number of species for most sites. In terms of land use, there was no clear pattern in the differences between the identification methods.

### 3.2 Detection Frequency per Species and Method

The number of sites where each earthworm species was detected by each identification method is depicted in Figure 3. *Allolobophora chlorotica* , *Aporrectodea caliginosa* , and *Aporrectodea rosea* were detected most frequently with all three methods. *Bimastos rubidus* and *Dendrobaena octaedra* were detected more frequently by eDNA metabarcoding than by the other two methods. In contrast, *Lumbricus terrestris* and both *Octolasion* species were detected less frequently with eDNA metabarcoding. Epigeic species (representatives of the genus *Dendrobaena* Eisen, 1873, *Bimastos rubidus*, *Lumbricus castaneus* and *Lumbricus rubellus* ) were not detected at some sites based on morphological identification. Comparing the results of a single method with the results of all three methods combined, comDNA metabarcoding showed the highest reliability for the detection of species, as the number of sites where the respective species were detected using this method was closest to the total number of sites where the species were detected using all identification methods.

### 3.3 Principal Coordinates Analysis

**FIGURE 5:**
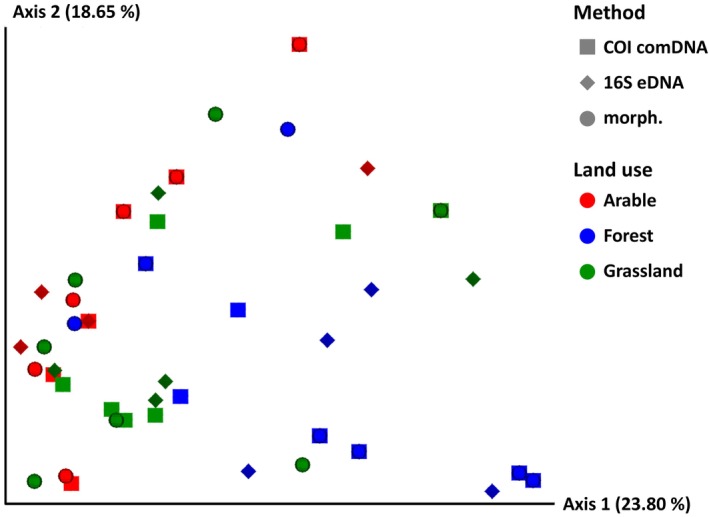
Principal coordinates analysis based on the similarity (Jaccard coefficient) between sites and identification methods regarding earthworm species composition (presence/absence). The axes represent the first and second principal coordinates, respectively. The percentages represent the contribution of the principal coordinates to explaining the total variation in the dataset.

The PCoA plot based on the Jaccard similarity index showed a continuous gradient in the species diversity per site from arable land to forest sites with a strong overlap in some cases (Figure 5). The grassland sites are located between the two other land use types. There is no obviously different pattern between the three identification methods. This means that all three identification methods can differentiate between sites with different species compositions, although the degree of separation between the sites may be different for each identification method.

### 3.4 Correlation Between the Relative Abundance and Biomass of Species and the Relative Number of eDNA Metabarcoding Reads

There was a weak, but statistically significant positive correlation between the proportion of sequencing reads obtained by eDNA metabarcoding and the relative abundance of species morphologically identified at each sampling site (*R* = 0.66, *p* = 1.4e‐06; see Appendix S4). A similar correlation was observed between the relative biomass and the eDNA metabarcoding reads (*R* = 0.67, *p* = 1.1e‐06; see Appendix S4). However, many data points lie outside the 95%‐confidence interval of the correlation line (data not shown) indicating that these correlations are unsuitable for predicting the relative abundance of species or biomass from the relative number of sequencing reads.

**FIGURE 6:**
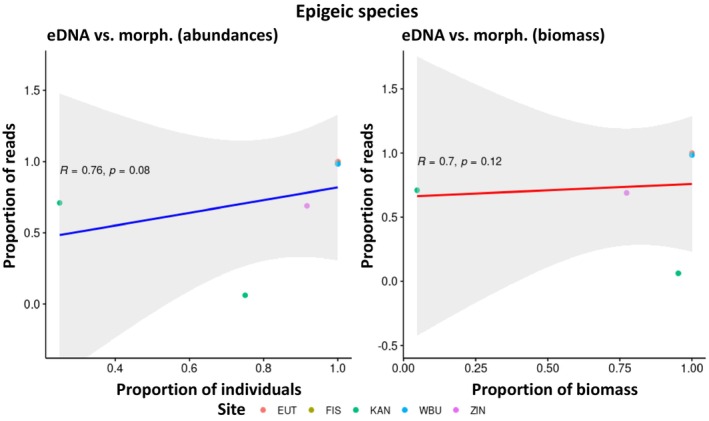
No statistically significant correlation between the relative abundance and biomass of epigeic species and the relative number of eDNA metabarcoding reads (see Table 1 for site abbreviations).

**FIGURE 7:**
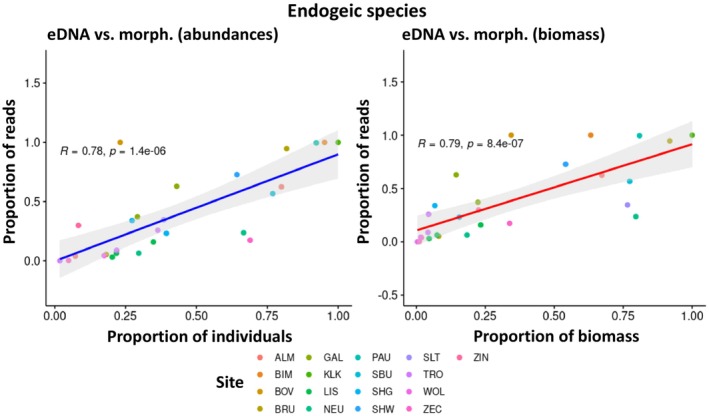
No statistically significant correlation between the relative abundance and biomass of anecic species and the relative number of eDNA metabarcoding reads (see Table 1 for site abbreviations).

**FIGURE 8:**
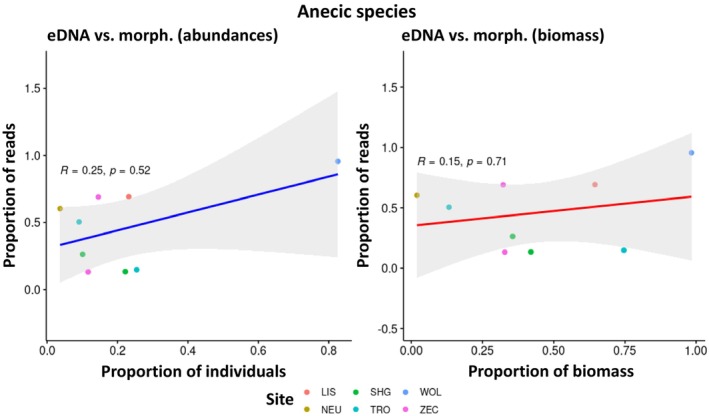
Strong and statistically significant positive correlation between the relative abundance and biomass of endogeic species and the relative number of eDNA metabarcoding reads (see Table 1 for site abbreviations).

In addition, such correlations were carried out for the five most frequently detected species: *Allolobophora chlorotica* , *Aporrectodea caliginosa* , *Aporrectodea longa* , *Aporrectodea rosea* , and *Lumbricus terrestris* (see Appendix S4). Interestingly, the results for the endogeic species *A. chlorotica* show a strong and statistically significant positive correlation between relative abundance (*R* = 1.00, *p* = 0.0028) and biomass (*R* = 0.89, *p* = 0.033) and the proportion of eDNA metabarcoding reads. In contrast, in *A. caliginosa* , also an endogeic species, there was only a very weak and only narrowly statistically significant correlation between the relative abundance and the eDNA metabarcoding reads (*R* = 0.55, *p* = 0.044). For relative biomass, the correlation was equally weak and not statistically significant (*R* = 0.53, *p* = 0.053). For the endogeic species *A. rosea* , on the other hand, the correlation was strong and statistically significant for relative abundance (*R* = 0.86, *p* = 0.024) and clearly visible, although not statistically significant, for relative biomass (*R* = 0.75, *p* = 0.066). Looking at the anecic species, there was no correlation between the relative abundance and biomass of *A. longa* and the number of eDNA metabarcoding reads (*R* = 0.40, *p* = 0.75 for both), which was equally true for *L. terrestris* (abundance: *R* = 0.10, *p* = 0.95; biomass: *R* = 0.30, *p* = 0.68). Thus, there were indications that the correlations might be stronger in endogeic species than in anecic species, which was subsequently investigated in more detail by calculating the correlations for the species summarized according to their ecological groups. The result confirmed the assumption: while no statistically significant correlations were found for epigeic (Figure 6) and anecic species (Figure 8), a strong and statistically significant positive correlation between the relative abundance and biomass and the proportion of eDNA metabarcoding reads was found for endogeic species (Figure 7).

## 4 Discussion

### 4.1 Earthworm Method Comparison

The three different identification methods employed fundamentally different pipelines to achieve the same goal, a final list of species for each investigated site. Each identification method was adapted individually to maximize the accuracy of species detection and identification. This included the sampling approach, the DNA extraction methods, as well as the choice of different molecular markers for comDNA and eDNA. Hence, what can be directly compared are the final lists of species obtained with the different methods. The results of this comparison showed that comDNA detected more species (both on average and at maximum) than eDNA or morphological identification. In contrast, eDNA, on average, detected a similar number of species as morphological identification. It should be noted here that the same number does not necessarily mean that the same species were detected. However, some species appear to have a higher or lower probability of being detected by eDNA than others. The abundance, behavior, or size of the species could explain the differences in detection frequency per species (Figure 3) and in the detected number of species per site (Figure 4). The endogeic species *Allolobophora chlorotica* , *Aporrectodea caliginosa* , and *Aporrectodea rosea* were those with the highest abundance. These species are known for their horizontal burrowing activity, creating tunnel networks within the soil resulting in the deposition of eDNA throughout the soil matrix. They primarily feed on soil organic matter, further contributing to the dispersal of eDNA through their digestive processes. Their reproduction strategies, involving laying cocoons within the soil, can further increase the amount of eDNA in their immediate environment, making them more detectable in soil samples compared to other ecological groups of earthworms, such as epigeic or anecic species (Bouché 1977). The abundance of the endogeic species belonging to the genus *Octolasion* Örley, 1885, that were less frequently identified by eDNA was very low compared to other endogeic species. In contrast, *Bimastos rubidus* and *Dendrobaena octaedra* were more frequently detected with eDNA. These small epigeic species could have been overlooked during hand‐sorting, while whole individuals, especially juveniles, could be present in the eDNA samples. The eDNA of vertical burrowers such as *Lumbricus terrestris* , on the other hand, could be very unevenly distributed in the soil, especially in their vertical burrows and their casts at their exits on the soil surface. Therefore, they were less likely to be detected by eDNA metabarcoding due to the random distribution of sampling points. One way to counteract this effect would be to take some of the eDNA samples specifically where superficially recognizable traces of deep‐burrowing earthworms are visible, as well as the targeted sampling of microhabitats such as dead wood, dung heaps, or moss cushions for the complete detection of epigeic species. However, this approach might stand in the way of the standardization of sampling that is generally desirable and requires the sampling personnel to be more highly qualified than in the case of uniform and observation‐independent sampling. Such methodological issues should be discussed in further developing and standardizing future sampling methods. Summarizing, we found that eDNA offers advantages in detecting epigeic species (in particular very small‐sized ones that might have been overlooked during hand‐sorting), while anecic species are more reliably detected by morphological and comDNA identification. Endogeic species are well‐detected by all identification methods provided they have a sufficient abundance. ComDNA can identify individuals to species level that are difficult to identify morphologically, for example, juveniles, cryptic species, and fragments. All three identification methods can differentiate between sites with different species composition; the degree of separation can vary depending on the identification method (Figure 5). The relative proportion of eDNA reads shows potential as a surrogate of relative abundance/biomass for endogeic but not for epigeic and anecic species (Figures 6, 7, 8). It would be desirable to conduct additional quantitative investigations in future studies, for example, how site and soil properties relate to the molecular diversity of earthworms and the comparative performance of each identification method. The current study was not designed to answer these questions, and the investigated number of sites does not support robust analyses in this regard.

Techniques of species identification based on eDNA are already used in environmental studies, for example, to assess land use effects on soil biodiversity (Brunetti et al. 2024; Cuartero et al. 2025). However, only a few studies have attempted to make a direct comparison of traditional and DNA‐based earthworm identification methods thus far. In their seminal study in the French Alps, Bienert et al. (2012) investigated two sites (with two plots, each), an undisturbed woodland and a maintained meadow, by hand‐sorting of 0.5 m^2^ sample plots with morphological identification of the collected earthworms and by metabarcoding of eDNA extracted from eight soil cores per plot taken from two soil layers (0–20 cm; 20–40 cm) in the vicinity of the hand‐sorting spots. They observed that epigeic species *Lumbricus friendi* and *Lumbricus castaneus* were missed by eDNA metabarcoding while endogeic species *A. chlorotica* and *Octolasion tyrtaeum* were missed by hand‐sorting. This effect was not found in the present study, which can be attributed to differences in the sampling approach. As recommended by Bienert et al. (2012) to increase the probability of detecting epigeic species, in the present study more individual soil cores, including the topsoil, were taken for eDNA metabarcoding and eDNA was extracted from several kilos of soil. In addition, unlike Bienert et al. (2012) we used AITC to extract earthworm individuals that might have withdrawn to deeper soil layers due to the mechanical disturbance during soil excavation.

Pansu et al. (2015) sampled plots of old beech coppice, young spruce plantation and pasture in the Northern French Alpes. Classical earthworm sampling was performed like in the present study by combined AITC extraction and hand‐sorting in June 2012 and October 2013 and eDNA metabarcoding analyses were performed from soil cores taken in October 2013. An alternative sampling approach with a higher number of individual soil cores per composite sample (100 vs. 10) across a regular grid (100 × 100 m vs. 0.5 m^2^ plots) was also applied to a subset of the plots in October 2011. Extraction of eDNA from soil using saturated phosphate buffer and the ‘NucleoSpin Soil’ kit likewise followed the same method as in the present contribution. A total of 13 species were identified morphologically. While eDNA detected 16 molecular operational units (MOTUs) and 31 with the alternative sampling approach, only seven and nine of these MOTUs could be assigned to the species level, respectively, due to incomplete reference databases. Comparability of the two identification methods and the results of the present study was hampered by the different sampling time points, the distance between the sampling spots for morphological identification and eDNA metabarcoding as well as a high local spatial heterogeneity.

Llanos et al. (2023) investigated the earthworm community of four arable fields with temporary grass‐clover ley strips sown in West Yorkshire, United Kingdom, by traditional earthworm hand‐sorting (eight soil pits of 18 × 18 × 15 cm per site) and morphological identification and eDNA metabarcoding (24 soil cores of 5 × 5 × 15 cm per site, two different 16S rDNA primer pairs). The authors found that while both traditional hand‐sorting with morphological identification and the eDNA method identified the same eight earthworm species overall, the mean number of species per sampling spot was significantly higher for eDNA than traditional hand‐sorting. This was, however, in large part due to the lack of anecic species identified in the hand‐sorting approach, in contrast to our observations where the opposite was the case. Unlike in the present study, Llanos et al. (2023) did not apply any chemical extraction method to expel anecic worms that may have fled to deeper soil layers due to the disturbance caused by the soil excavation. They also observed that eDNA performed comparatively worse for the epigeic species *Satchellius mammalis* (Savigny, 1826) and speculated that this might be due to faster eDNA degradation in the topsoil.

Lilja et al. (2023) sampled two experimental agricultural sites with three different fertiliser treatments in Denmark using hand‐sorting (two soil blocks of 20 × 20 × 30 cm per experimental plot) with conventional morphology‐based identification (combined with Sanger sequencing where necessary) and eDNA metabarcoding (15 soil cores of 20 cm depth and 2.5 cm diameter per plot) using two different DNA extraction kits. eDNA metabarcoding detected a higher total number of earthworm species (eight) than morphological identification (five). However, as in Llanos et al. (2023), no chemical extraction fluid was applied, making the detection of anecic species such as *Lumbricus terrestris* by conventional sampling less likely, and that was indeed one of the three species not identified by conventional sampling and identification.

### 4.2 Evaluation of the Methods for Routine Operation in Continuous Soil Monitoring

As the data collected as part of this contribution show, it is possible to achieve the general objectives of long‐term soil monitoring using genetic identification methods. The current biological state of the soil, as indicated by the respective earthworm community, can be described using these methods. This mostly applies to qualitative parameters such as the presence/absence of species but can also be applied to quantitative parameters (number of species, abundance) to a limited extent. Quantitatively, the number of species per site and the number of metabarcoding reads per species or ASV/taxon can be specified. The results indicate that for earthworms, conclusions can also be drawn from this regarding relative abundance or biomass. This should therefore be investigated in more detail in future, specially tailored research projects. Qualitatively, the species composition can be specified for the sites to be analysed, possibly by assigning species to different ecological groups. Site diversity could be characterised using suitable diversity indices.

The quantitative and qualitative parameters collected at a site can be used to monitor long‐term changes in soil biology, provided that the same standardised methods are used (e.g., ISO) to make the data collected at different times and at different sites comparable. The rapidly advancing technological development in high‐throughput DNA sequencing should not be an obstacle to standardisation. Methods such as spike‐in DNA, model‐based pipeline noise estimation, and unique molecular identifiers (Luo et al. 2023), as well as specimen photography, body measurements, and correction factors (Sickel et al. 2023), provide robust quantitative data. While these advanced methods may incur additional costs, they enhance the accuracy and reliability of data. Predictions about future developments are feasible with the genetic identification methods, given sufficient knowledge about the drivers of such developments, although this also applies equally to the classical morphological methods.

Genetic identification methods can thus contribute to a sound data basis for environmental and environmental policy decision‐making processes. Reliable soil health indicators/reference values are necessary for some such decisions (e.g., European Commission 2021), and the use of DNA metabarcoding in this context is recommended (Pieper et al. 2023). All three identification methods used in this project provide the tools to develop such soil health indices, for example, in the form of reference values based on earthworm diversity. Quantitative measurements of soil fauna are also required, for example, for assessing the effects of anthropogenic stressors on earthworm abundance and on ecosystem functions performed by earthworms (Pieper et al. 2023) or in the scope of a next generation, systems‐based prospective environmental risk assessment of pesticides linked to monitoring (Sousa et al. 2022). Here, molecular methods have only allowed for relative comparisons, for example, based on the number of metabarcoding reads. In addition, genetic methods are currently not considered sufficient to register new species or genotypes when the corresponding sequences are not yet available in the reference database, together with a valid species name. Therefore, in certain cases, but especially when genetic methods are used for the first time at a site, classical methods, including sampling and morphological identification, and DNA‐based methods, should be used in combination, with comDNA metabarcoding serving as a bridge between morphological and eDNA‐based data:

- to link historical (i.e., morphology‐based) with future (DNA‐based) data;
- for decision‐making processes that require quantitative data;
- for a comprehensive assessment of diversity at sites with unknown species composition.

Regarding the costs incurred for the application of genetic identification methods, it can be stated that DNA‐based methods, depending on the study design, potentially generate considerably lower costs than the classical morphological sampling and identification methods (Ji et al. 2013), especially when using eDNA and with higher site/sample numbers. We attempted to estimate this based on the number of sites and samples processed in the scope of this contribution (data not shown). While the labour required for the classical sampling and morphological earthworm identification increases linearly with the number of samples to be processed, the costs per sample for genetic work decrease significantly with higher sample numbers. This is partly due to the high fixed costs incurred for a single sequencing run of an Illumina MiSeq PE300, in which up to 600 samples can be processed in parallel. Due to the high workload of preparing a comDNA sample, as the animals must first be extracted in the field, the total costs of earthworm identification using comDNA metabarcoding are similar (about 90%) to those of morphological identification. On the other hand, earthworm identification using only eDNA generated about 30% of the costs of classical sampling and morphological identification. In addition, eDNA metabarcoding offers the great advantage that it can be used to simultaneously record many different groups of soil fauna (Bienert et al. 2012; Taberlet et al. 2018).

## 5 Conclusions

Based on the results of this work, it can be concluded that genetic identification methods using comDNA and eDNA metabarcoding are suitable for achieving the objectives of long‐term soil monitoring and can be integrated into routine earthworm monitoring. These methods, particularly eDNA metabarcoding, are expected to play a crucial role in future soil biodiversity assessments, as evidenced by their application in the ‘Land Use and Land Cover Survey’ (LUCAS; Jones et al. 2021) and studies using eDNA identification of earthworms to assess land use effects on soil biodiversity (Brunetti et al. 2024; Cuartero et al. 2025). Recent advancements in enhancing eDNA data analysis for biomonitoring, as outlined by Stenger et al. (2025), further support the growing role of these methods in soil health assessments. This advancement will contribute to a new metric for soil biodiversity, raising the question of whether molecular methods will provide equivalent or even more reliable insights into soil health compared to classical approaches.

Molecular methods are already successfully applied in other environmental assessments, such as evaluating surface water quality using biological indices (Hering et al. 2018; Kuntke et al. 2020; Vivien et al. 2020). Thus, no fundamental barrier exists to their routine application in soil monitoring, provided key requirements are met (see also Ruppert et al. 2019):

- DNA reference databases must be comprehensive, well‐curated, and quality‐controlled to minimize errors in species identification.
- DNA‐based methods require standardisation (e.g., within ISO) from field sampling to data analysis to ensure consistency and reproducibility.
- Reliable threshold values and indices for soil health need to be developed based on community‐level data of soil organisms (Pieper et al. 2023), a goal implemented in research initiatives under the EU Soil Strategy for 2030 (European Commission 2021).

These requirements highlight the following research needs:

- Refinement of methodologies, including optimal sampling designs, number of composite eDNA samples, and DNA extraction/metabarcoding protocols.
- Development of correction factors to correlate earthworm abundance and biomass with sequencing read numbers, enabling more accurate quantification.
- Investigation of site and soil properties in relation to molecular diversity and the comparative efficacy of identification methods.
- Determination of eDNA availability based on soil properties and earthworm biological traits.
- Assessment of DNA retention time in soil under varying environmental conditions.
- Tracking and handling of possible primer‐associated bias during DNA amplification.
- Expansion, curation, and quality assurance of DNA reference databases.
- Taxonomic validation and formal description of newly discovered species to resolve discrepancies, integrating morphological characterisation and individuum‐based Sanger sequencing.
- Establishment of soil health threshold values and reference indices.
- Evaluation of additional soil animal groups (e.g., collembolans, enchytraeids, nematodes, mites, myriapods, isopods) for future monitoring programs.

In conclusion, genetic identification methods, particularly eDNA metabarcoding, offer a scalable, cost‐effective, and reliable approach for monitoring earthworm communities and broader soil biodiversity. Future research should focus on refining quantitative metrics, improving reference databases, and integrating these methodologies into standardised frameworks to maximise their utility for environmental management and policy development.

## Author Contributions

**Stephan Jänsch:** conceptualization (equal), data curation (equal), funding acquisition (supporting), methodology (equal), project administration (equal), visualization (supporting), writing – original draft (lead), writing – review and editing (equal). **Daniela Alves:** conceptualization (supporting), data curation (equal), investigation (equal), methodology (equal), project administration (supporting), visualization (supporting), writing – original draft (supporting), writing – review and editing (supporting). **Luís Cunha:** conceptualization (supporting), data curation (supporting), methodology (supporting), visualization (supporting), writing – original draft (supporting), writing – review and editing (equal). **Paul Henning Krogh:** conceptualization (equal), funding acquisition (supporting), investigation (equal), methodology (equal), project administration (supporting), resources (supporting), writing – review and editing (equal). **Tiago Natal‐da‐Luz:** conceptualization (equal), data curation (supporting), investigation (supporting), methodology (equal), project administration (supporting), visualization (supporting), writing – original draft (supporting), writing – review and editing (equal). **Verónica Rojo:** data curation (equal), formal analysis (lead), visualization (lead), writing – review and editing (equal). **Jörg Römbke:** conceptualization (equal), funding acquisition (lead), investigation (supporting), methodology (equal), project administration (equal), resources (lead), supervision (equal). **Rumakanta Sapkota:** conceptualization (supporting), investigation (supporting), methodology (supporting), resources (supporting), writing – review and editing (equal). **Adam Scheffczyk:** conceptualization (equal), data curation (equal), investigation (lead), methodology (equal), supervision (equal). **Rüdiger M. Schmelz:** conceptualization (equal), funding acquisition (supporting), investigation (supporting), methodology (equal), project administration (supporting), validation (equal), visualization (supporting), writing – original draft (equal), writing – review and editing (equal). **Letícia Scopel:** conceptualization (supporting), data curation (equal), investigation (equal), methodology (equal), project administration (supporting), visualization (supporting), writing – original draft (supporting), writing – review and editing (supporting). **José Paulo Sousa:** conceptualization (equal), data curation (supporting), funding acquisition (supporting), investigation (supporting), methodology (supporting), project administration (supporting), visualization (supporting), writing – original draft (supporting), writing – review and editing (equal). **Joaquín Vierna:** funding acquisition (supporting), project administration (supporting), resources (equal), supervision (equal). **Antón Vizcaíno:** investigation (equal), methodology (equal), writing – review and editing (equal).

## Conflicts of Interest

The authors declare no conflicts of interest.

## Supporting information


Appendices S1.

Appendices S2.

Appendices S3.

Appendices S4.

## Data Availability

The raw sequencing data are deposited in the NCBI SRA database under accession number BioProject: PRJNA1134652 and can be reviewed at https://www.ncbi.nlm.nih.gov/bioproject/PRJNA1134652.
