# Correction to “Comparison of Morphological and DNA‐Based Identification Methods to Assess Earthworm (Clitellata: Lumbricidae) Diversity at 25 Permanent Soil Monitoring Sites in Germany”

**DOI:** 10.1002/ece3.73577

**Published:** 2026-04-20

Jänsch, S., Alves, D., Cunha, L., Krogh, P.H., Natal‐da‐Luz, T., Rojo, V., Römbke, J., Sapkota, R., Scheffczyk, A., Schmelz, R.M., Scopel, L., Sousa, J.P., Vierna, J. and Vizcaíno, A. 2025 “Comparison of Morphological and DNA‐Based Identification Methods to Assess Earthworm (Clitellata: Lumbricidae) Diversity at 25 Permanent Soil Monitoring Sites in Germany.” *Ecology and Evolution*, 15: e71155. https://doi.org/10.1002/ece3.71155.

The figure captions for Figures 7 and 8 were swapped. The data contained in Figure 7 relate to endogeic and the data in Figure 8 to anecic species, not vice versa.

The correct captions are shown below.

FIGURE 7: Strong and statistically significant positive correlation between the relative abundance and biomass of endogeic species and the relative number of eDNA metabarcoding reads (see Table 1 for site abbreviations).

FIGURE 8: No statistically significant correlation between the relative abundance and biomass of anecic species and the relative number of eDNA metabarcoding reads (see Table 1 for site abbreviations).

We apologize for this error.

