# Supplementary material for: Comparison of Morphological and DNA‐Based Identification Methods to Assess Earthworm (Clitellata: Lumbricidae) Diversity at 25 Permanent Soil Monitoring Sites in Germany
Source: Ecol Evol. 2025 Mar 30;15(4):e71155. doi: 10.1002/ece3.71155 (PMC11955243; doi:10.1002/ece3.71155)
Supplement: Supplementary file 1 — Appendices S1. [file ECE3-15-e71155-s001.docx]

**Appendix S1: Adjusting of species lists for method comparison, specific example of the genus *Aporrectodea***

At the Almesbach Stallkoppel (ALM) site, a COI-ASV with a low number of comDNA metabarcoding reads was assigned to the species *Aporrectodea terrestris* (Savigny, 1826), whereas it was not detected in the morphological identification. This is presumably related to the unclear species status of this taxon, especially regarding the distinction from *A. longa*. According to Porco et al. (2018), based on pairwise comparisons of the COI molecular OTUs, *A. longa* and *A. terrestris* could at least partially lack a reproductive barrier and therefore not be considered molecular OTUs at the species level. Therefore, for *A. terrestris* it was decided to assign this ASV to *A. longa* instead. At some sites, some ASVs were assigned to the taxa *Aporrectodea trapezoides* (Dugés, 1828) or *A. trapezoides* L1 in the comDNA and eDNA metabarcoding analyses, but not in the morphological identification. After a more detailed comparison of the nucleotide identity values between the COI ASVs assigned to *A. caliginosa* and *A. trapezoides* at the ALM and Zeckerin (ZEC) sites, it became clear that there are several strongly divergent COI lineages within this species complex, which apparently also include *A. trapezoides* (Table S1.1, Table S1.2). It was, therefore, decided to treat these taxa as other COI lineages of *A. caliginosa*. This approach to classification is also consistent with the conclusions of Briones (1996). Accordingly, *A. trapezoides* is a subspecies of *A. caliginosa*, and since no differentiation according to subspecies was made in the morphological identification in the context of this project, it is justified to group *A. trapezoides* together with *A. caliginosa*.

Table S1.1: Nucleotide identity matrix between COI ASV sequences at the Almesbach Stallkoppel (ALM) site assigned to *Aporrectodea caliginosa* and *A. trapezoides*

|  | *A. caliginosa* | *A. caliginosa* L2 | *A. caliginosa* L3 | *A. trapezoides* | *A. trapezoides* L1 |
| --- | --- | --- | --- | --- | --- |
| *A. caliginosa* |  | 79.87 % – 99.04 % * | 78.91 % – 85.94 % | 82.11 % – 84.66 % | 77.64 % –  83.60 % |
| *A. caliginosa* L2 | 79.87 % – 99.04 % * |  | 85.21 % | 82.75 % – 83.39 % | 83.28 % –  83.60 % |
| *A. caliginosa* L3 | 78.91 % – 85.94 % | 85.21 % |  | 80.83 % – 83.39 % | 83.39 % –  83.71 % |
| *A. trapezoides* | 82.11 % – 84.66 % | 82.75 % – 83.39 % | 80.83 % – 83.39 % |  | 83.39 % –  85.30 % |
| *A. trapezoides* L1 | 77.64 % – 83.60 % | 83.28 % – 83.60 % | 83.39 % – 83.71 % | 83.39 % – 85.30 % |  |

* Some reference sequences labelled simply as "*A. caliginosa*" probably belong to lineage L2.

Table S1.2: Nucleotide identity matrix between COI ASV sequences at the Zeckerin site (ZEC) assigned to *Aporrectodea caliginosa* and *A. trapezoides*

|  | *A. caliginosa* | *A. caliginosa* L3 | *A. trapezoides* L1 |
| --- | --- | --- | --- |
| *A. caliginosa* |  | 95.85 % – 99.68 % * | 81.15 % – 83.71 % |
| *A. caliginosa* L3 | 95.85 % – 99.68 % * |  | 81.47 % |
| *A. trapezoides* L1 | 81.15 % – 83.71 % | 81.47 % |  |

* Some reference sequences labelled simply as "*A. caliginosa*" probably belong to lineage L3.

**References**

Briones, M. J. I. (1996). A taxonomic revision of the *Allolobophora caliginosa* complex (Oligochaeta, Lumbricidae): a preliminary study. *Canadian Journal of Zoology*, 74(2), 240-244. <https://doi.org/10.1139/z96-030>

Porco, D., Chang, C.-H., Dupont, L., James, S., Richard, B., & Decaëns, T. (2018). A reference library of DNA bar-codes for the earthworms from Upper Normandy: Biodiversity assessment, new records, potential cases of cryptic diversity and ongoing speciation. *Applied Soil Ecology*, 124, 362-371. <https://doi.org/10.1016/j.apsoil.2017.11.001>
