# Supplementary material for: Comparison of Morphological and DNA‐Based Identification Methods to Assess Earthworm (Clitellata: Lumbricidae) Diversity at 25 Permanent Soil Monitoring Sites in Germany
Source: Ecol Evol. 2025 Mar 30;15(4):e71155. doi: 10.1002/ece3.71155 (PMC11955243; doi:10.1002/ece3.71155)
Supplement: Supplementary file 2 — Appendices S2. [file ECE3-15-e71155-s002.docx]

**Appendix S2: Results of the three identification methods**

Table S2.1: Abundance of earthworms [ind/m²] at the sampled arable sites

| Taxon | BIM | GHU | KLK | LIS | NEU | SLT | TRO | WOL |
| --- | --- | --- | --- | --- | --- | --- | --- | --- |
| *Aporrectodea* sp. sensu lato^1^ | 14.4 | 22.4 | 12.0 | 80.8 | 37.6 | 32.0 | 61.6 | 2.4 |
| *Allolobophora chlorotica* | 0.0 | 0.0 | 0.0 | 12.0 | 12.8 | 0.0 | 0.8 | 0.0 |
| *Aporrectodea caliginosa* | 1.6 | 35.2 | 5.6 | 19.2 | 28.8 | 0.8 | 9.6 | 0.0 |
| *Aporrectodea longa* | 0.0 | 0.0 | 0.0 | 12.8 | 0.0 | 0.0 | 4.0 | 0.0 |
| *Aporrectodea rosea* | 0.0 | 3.2 | 0.0 | 11.2 | 0.0 | 0.0 | 16.0 | 0.8 |
| *Lumbricus* spp. | 0.0 | 1.6 | 0.0 | 0.0 | 1.6 | 4.0 | 15.2 | 2.4 |
| *Lumbricus rubellus* | 0.0 | 0.0 | 0.0 | 0.0 | 0.0 | 2.4 | 0.0 | 0.0 |
| *Lumbricus terrestris* | 0.8 | 5.6 | 0.0 | 0.0 | 0.0 | 0.0 | 11.2 | 12.8 |
| *Octolasion* spp. | 0.0 | 0.0 | 0.0 | 0.0 | 0.0 | 0.0 | 1.6 | 0.0 |
| *Octolasion tyrtaeum* | 0.0 | 0.0 | 0.0 | 0.0 | 0.0 | 0.0 | 0.8 | 0.0 |
| Not determinable | 0.0 | 13.6 | 0.8 | 20.8 | 6.4 | 4.0 | 12.0 | 1.6 |
| Tanylobous species | 0.8 | 7.2 | 0.0 | 0.0 | 1.6 | 6.4 | 26.4 | 15.2 |
| Epilobous species | 16.0 | 60.8 | 17.6 | 136.0 | 79.2 | 32.8 | 94.4 | 3.2 |
| Epigeic adults | 0.0 | 0.0 | 0.0 | 0.0 | 0.0 | 2.4 | 0.0 | 0.0 |
| Endogeic adults | 1.6 | 38.4 | 5.6 | 42.4 | 41.6 | 0.8 | 27.2 | 0.8 |
| Anecic adults | 0.8 | 5.6 | 0.0 | 12.8 | 0.0 | 0.0 | 15.2 | 12.8 |
| Sum of adults | 2.4 | 44.0 | 5.6 | 55.2 | 41.6 | 3.2 | 42.4 | 13.6 |
| Sum of juveniles | 14.4 | 24.0 | 12.0 | 80.8 | 39.2 | 36.0 | 78.4 | 4.8 |
| **Total abundance** | **16.8** | **81.6** | **18.4** | **156.8** | **87.2** | **43.2** | **132.8** | **20.0** |
| **No. of species** | **2** | **3** | **1** | **4** | **3** | **2** | **6** | **2** |

¹ Not differentiated between the closely related genera *Aporrectodea* and *Allolobophora*

Table S2.2: Biomass of earthworms [g/m²] at the sampled arable sites

| Taxon | BIM | GHU | KLK | LIS | NEU | SLT | TRO | WOL |
| --- | --- | --- | --- | --- | --- | --- | --- | --- |
| *Aporrectodea* sp. sensu lato^1^ | 2.26 | 5.68 | 1.95 | 21.80 | 3.48 | 5.51 | 7.63 | 0.41 |
| *Allolobophora chlorotica* | 0.00 | 0.00 | 0.00 | 2.47 | 1.80 | 0.00 | 0.23 | 0.00 |
| *Aporrectodea caliginosa* | 0.75 | 24.18 | 2.22 | 7.68 | 7.79 | 0.82 | 2.41 | 0.00 |
| *Aporrectodea longa* | 0.00 | 0.00 | 0.00 | 21.20 | 0.00 | 0.00 | 7.47 | 0.00 |
| *Aporrectodea rosea* | 0.00 | 0.66 | 0.00 | 1.53 | 0.00 | 0.00 | 2.53 | 0.08 |
| *Lumbricus* spp. | 0.00 | 1.02 | 0.00 | 0.00 | 0.20 | 0.94 | 8.28 | 2.44 |
| *Lumbricus rubellus* | 0.00 | 0.00 | 0.00 | 0.00 | 0.00 | 1.00 | 0.00 | 0.00 |
| *Lumbricus terrestris* | 1.75 | 11.11 | 0.00 | 0.00 | 0.00 | 0.00 | 42.07 | 26.83 |
| *Octolasion* spp. | 0.00 | 0.00 | 0.00 | 0.00 | 0.00 | 0.00 | 0.98 | 0.00 |
| *Octolasion tyrtaeum* | 0.00 | 0.00 | 0.00 | 0.00 | 0.00 | 0.00 | 0.68 | 0.00 |
| Not determinable | 0.00 | 4.33 | 0.09 | 4.94 | 1.28 | 0.77 | 1.20 | 0.68 |
| Tanylobous species | 1.75 | 12.14 | 0.00 | 0.00 | 0.20 | 1.94 | 50.35 | 29.27 |
| Epilobous species | 3.02 | 30.53 | 4.18 | 54.68 | 13.07 | 6.34 | 21.93 | 0.49 |
| Epigeic adults | 0.00 | 0.00 | 0.00 | 0.00 | 0.00 | 1.00 | 0.00 | 0.00 |
| Endogeic adults | 0.75 | 24.85 | 2.22 | 11.68 | 9.59 | 0.82 | 5.85 | 0.08 |
| Anecic adults | 1.75 | 11.11 | 0.00 | 21.20 | 0.00 | 0.00 | 49.54 | 26.83 |
| Sum of adults | 2.50 | 35.96 | 2.22 | 32.88 | 9.59 | 1.82 | 55.39 | 26.91 |
| Sum of juveniles | 2.26 | 6.70 | 1.95 | 21.80 | 3.68 | 6.45 | 16.89 | 2.85 |
| **Total biomass** | **4.77** | **46.99** | **4.26** | **59.62** | **14.55** | **9.04** | **73.48** | **30.44** |

¹ Not differentiated between the closely related genera *Aporrectodea* and *Allolobophora*

Table S2.3: Abundance of earthworms [ind/m²] at the sampled grassland sites

| Taxon | ALM | BRU | GAL | KUM | PAU | SHG | ZEC | ZIN |
| --- | --- | --- | --- | --- | --- | --- | --- | --- |
| *Aporrectodea* sp. sensu lato^1^ | 22.4 | 2.4 | 18.4 | 2.4 | 0.8 | 12.0 | 132.8 | 5.6 |
| *Allolobophora chlorotica* | 0.0 | 1.6 | 3.2 | 0.0 | 28.8 | 31.2 | 0.0 | 0.0 |
| *Aporrectodea caliginosa* | 35.2 | 7.2 | 16.8 | 1.6 | 0.0 | 0.0 | 56.8 | 4.0 |
| *Aporrectodea longa* | 0.0 | 0.0 | 4.0 | 0.0 | 0.8 | 17.6 | 12.0 | 0.0 |
| *Aporrectodea rosea* | 3.2 | 0.0 | 24.8 | 1.6 | 0.0 | 21.6 | 4.0 | 0.0 |
| *Lumbricus* spp. | 1.6 | 0.0 | 7.2 | 0.8 | 0.8 | 4.0 | 37.6 | 22.4 |
| *Lumbricus rubellus* | 0.0 | 0.0 | 2.4 | 0.0 | 0.8 | 0.0 | 0.0 | 21.6 |
| *Lumbricus terrestris* | 5.6 | 0.0 | 0.8 | 3.2 | 0.0 | 8.0 | 9.6 | 0.0 |
| *Octolasion cyaneum* | 0.0 | 0.0 | 0.8 | 1.6 | 0.0 | 0.0 | 0.0 | 0.0 |
| *Octolasion tyrtaeum* | 0.0 | 0.0 | 4.8 | 0.0 | 0.0 | 0.0 | 0.0 | 0.0 |
| *Proctodrilus antipae* | 0.0 | 0.0 | 0.0 | 0.0 | 0.0 | 0.8 | 0.0 | 0.0 |
| Not determinable | 13.6 | 1.6 | 14.4 | 0.0 | 0.8 | 12.8 | 43.2 | 4.0 |
| Tanylobous species | 7.2 | 0.0 | 10.4 | 4.0 | 1.6 | 12.0 | 47.2 | 44.0 |
| Epilobous species | 60.8 | 11.2 | 72.8 | 7.2 | 30.4 | 83.2 | 205.6 | 9.6 |
| Epigeic adults | 0.0 | 0.0 | 2.4 | 0.0 | 0.8 | 0.0 | 0.0 | 21.6 |
| Endogeic adults | 38.4 | 8.8 | 50.4 | 4.8 | 28.8 | 53.6 | 60.8 | 4.0 |
| Anecic adults | 5.6 | 0.0 | 4.8 | 3.2 | 0.8 | 25.6 | 21.6 | 0.0 |
| Sum of adults | 44.0 | 8.8 | 57.6 | 8.0 | 30.4 | 79.2 | 82.4 | 25.6 |
| Sum of juveniles | 24.0 | 2.4 | 25.6 | 3.2 | 1.6 | 16.0 | 170.4 | 28.0 |
| **Total abundance** | **81.6** | **12.8** | **97.6** | **11.2** | **32.8** | **108.0** | **296.0** | **57.6** |
| **No. of species** | **3** | **2** | **8** | **4** | **3** | **5** | **4** | **2** |

¹ Not differentiated between the closely related genera *Aporrectodea* and *Allolobophora*

Table S2.4: Biomass of earthworms [g/m²] at the sampled grassland sites

| Taxon | ALM | BRU | GAL | KUM | PAU | SHG | ZEC | ZIN |
| --- | --- | --- | --- | --- | --- | --- | --- | --- |
| *Aporrectodea* sp. sensu lato^1^ | 5.68 | 0.28 | 2.04 | 0.28 | 0.07 | 2.42 | 33.83 | 1.98 |
| *Allolobophora chlorotica* | 0.00 | 0.35 | 0.78 | 0.00 | 7.98 | 9.74 | 0.00 | 0.00 |
| *Aporrectodea caliginosa* | 24.18 | 3.98 | 6.81 | 0.54 | 0.00 | 0.00 | 21.93 | 4.01 |
| *Aporrectodea longa* | 0.00 | 0.00 | 6.79 | 0.00 | 1.06 | 26.22 | 20.89 | 0.00 |
| *Aporrectodea rosea* | 0.66 | 0.00 | 4.40 | 0.22 | 0.00 | 4.19 | 0.69 | 0.00 |
| *Lumbricus* spp. | 1.02 | 0.00 | 2.50 | 1.42 | 0.11 | 0.86 | 24.30 | 3.30 |
| *Lumbricus rubellus* | 0.00 | 0.00 | 2.16 | 0.00 | 0.71 | 0.00 | 0.00 | 10.41 |
| *Lumbricus terrestris* | 11.11 | 0.00 | 2.41 | 9.26 | 0.00 | 22.22 | 21.26 | 0.00 |
| *Octolasion cyaneum* | 0.00 | 0.00 | 1.17 | 1.88 | 0.00 | 0.00 | 0.00 | 0.00 |
| *Octolasion tyrtaeum* | 0.00 | 0.00 | 5.88 | 0.00 | 0.00 | 0.00 | 0.00 | 0.00 |
| *Proctodrilus antipae* | 0.00 | 0.00 | 0.00 | 0.00 | 0.00 | 0.07 | 0.00 | 0.00 |
| Not determinable | 4.33 | 0.13 | 1.86 | 0.00 | 0.30 | 5.07 | 15.49 | 0.83 |
| Tanylobous species | 12.14 | 0.00 | 7.07 | 10.67 | 0.82 | 23.08 | 45.57 | 13.71 |
| Epilobous species | 30.53 | 4.61 | 27.86 | 2.93 | 9.10 | 42.64 | 77.34 | 5.99 |
| Epigeic adults | 0.00 | 0.00 | 2.16 | 0.00 | 0.71 | 0.00 | 0.00 | 10.41 |
| Endogeic adults | 24.85 | 4.33 | 19.03 | 2.65 | 7.98 | 14.00 | 22.62 | 4.01 |
| Anecic adults | 11.11 | 0.00 | 9.20 | 9.26 | 1.06 | 48.43 | 42.15 | 0.00 |
| Sum of adults | 35.96 | 4.33 | 30.39 | 11.90 | 9.74 | 62.43 | 64.77 | 14.42 |
| Sum of juveniles | 6.70 | 0.28 | 4.54 | 1.70 | 0.18 | 3.29 | 58.14 | 5.28 |
| **Total biomass** | **46.99** | **4.74** | **36.79** | **13.60** | **10.22** | **70.79** | **138.39** | **20.54** |

¹ Not differentiated between the closely related genera *Aporrectodea* and *Allolobophora*

Table S2.5: Abundance of earthworms [ind/m²] at the sampled forest sites

| Taxon | BOV | EUT | FIS | KAN | MER | POS | SBU | SHW | WBU |
| --- | --- | --- | --- | --- | --- | --- | --- | --- | --- |
| *Aporrectodea* sp. sensu lato^1^ | 4.0 | 0.0 | 0.0 | 0.0 | 0.0 | 11.2 | 12.0 | 8.8 | 0.0 |
| *Aporrectodea caliginosa* | 2.4 | 0.0 | 0.0 | 0.0 | 0.0 | 1.6 | 8.0 | 14.4 | 0.0 |
| *Aporrectodea rosea* | 3.2 | 0.0 | 0.0 | 0.0 | 0.0 | 0.0 | 0.0 | 6.4 | 0.0 |
| *Dendrobaena* spp. | 0.0 | 0.0 | 0.0 | 0.8 | 0.0 | 0.0 | 0.0 | 0.0 | 20.0 |
| *Dendrobaena attemsi* | 0.0 | 0.0 | 0.0 | 0.0 | 0.0 | 0.0 | 0.0 | 0.0 | 3.2 |
| *Dendrobaena octaedra* | 0.0 | 0.0 | 0.0 | 3.2 | 0.0 | 0.0 | 0.0 | 0.0 | 0.0 |
| *Dendrodrilus rubidus* | 0.0 | 1.6 | 0.0 | 0.0 | 0.0 | 0.0 | 0.0 | 0.0 | 0.0 |
| *Lumbricus* spp. | 0.0 | 0.0 | 2.4 | 2.4 | 0.0 | 1.6 | 8.8 | 3.2 | 0.0 |
| *Lumbricus castaneus* | 0.0 | 0.0 | 0.0 | 0.0 | 0.0 | 0.0 | 2.4 | 0.0 | 0.0 |
| *Lumbricus rubellus* | 0.0 | 0.0 | 4.0 | 7.2 | 0.0 | 0.0 | 0.0 | 0.0 | 0.0 |
| *Lumbricus terrestris* | 0.0 | 0.0 | 0.0 | 0.0 | 0.0 | 0.8 | 0.0 | 0.8 | 0.0 |
| *Octolasion* spp. | 2.4 | 0.0 | 0.0 | 0.0 | 0.0 | 0.0 | 0.0 | 0.8 | 0.0 |
| *Octolasion cyaneum* | 2.4 | 0.0 | 0.0 | 0.0 | 0.0 | 0.0 | 0.0 | 0.8 | 0.0 |
| Not determinable | 1.6 | 0.0 | 0.0 | 3.2 | 0.0 | 0.0 | 0.8 | 2.4 | 0.0 |
| Tanylobous species | 0.0 | 0.0 | 6.4 | 9.6 | 0.0 | 2.4 | 11.2 | 4.0 | 0.0 |
| Epilobous species | 14.4 | 1.6 | 0.0 | 4.0 | 0.0 | 12.8 | 20.0 | 31.2 | 23.2 |
| Epigeic adults | 0.0 | 1.6 | 4.0 | 10.4 | 0.0 | 0.0 | 2.4 | 0.0 | 3.2 |
| Endogeic adults | 8.0 | 0.0 | 0.0 | 0.0 | 0.0 | 1.6 | 8.0 | 21.6 | 0.0 |
| Anecic adults | 0.0 | 0.0 | 0.0 | 0.0 | 0.0 | 0.8 | 0.0 | 0.8 | 0.0 |
| Sum of adults | 8.0 | 1.6 | 4.0 | 10.4 | 0.0 | 2.4 | 10.4 | 22.4 | 3.2 |
| Sum of juveniles | 6.4 | 0.0 | 2.4 | 3.2 | 0.0 | 12.8 | 20.8 | 12.8 | 20.0 |
| **Total abundance** | **16.0** | **1.6** | **6.4** | **16.8** | **0.0** | **15.2** | **32.0** | **37.6** | **23.2** |
| **No. of species** | **3** | **1** | **1** | **2** | **0** | **2** | **2** | **4** | **1** |

¹ Not differentiated between the closely related genera *Aporrectodea* and *Allolobophora*

Table S2.6: Biomass of earthworms [g/m²] at the sampled forest sites

| Taxon | BOV | EUT | FIS | KAN | MER | POS | SBU | SHW | WBU |
| --- | --- | --- | --- | --- | --- | --- | --- | --- | --- |
| *Aporrectodea* sp. sensu lato^1^ | 0.69 | 0.00 | 0.00 | 0.00 | 0.00 | 1.26 | 1.09 | 0.86 | 0.00 |
| *Aporrectodea caliginosa* | 3.49 | 0.00 | 0.00 | 0.00 | 0.00 | 0.46 | 4.13 | 4.70 | 0.00 |
| *Aporrectodea rosea* | 0.89 | 0.00 | 0.00 | 0.00 | 0.00 | 0.00 | 0.00 | 0.94 | 0.00 |
| *Dendrobaena* spp. | 0.00 | 0.00 | 0.00 | 0.05 | 0.00 | 0.00 | 0.00 | 0.00 | 0.67 |
| *Dendrobaena attemsi* | 0.00 | 0.00 | 0.00 | 0.00 | 0.00 | 0.00 | 0.00 | 0.00 | 0.13 |
| *Dendrobaena octaedra* | 0.00 | 0.00 | 0.00 | 0.40 | 0.00 | 0.00 | 0.00 | 0.00 | 0.00 |
| *Dendrodrilus rubidus* | 0.00 | 0.44 | 0.00 | 0.00 | 0.00 | 0.00 | 0.00 | 0.00 | 0.00 |
| *Lumbricus* spp. | 0.00 | 0.00 | 0.53 | 0.50 | 0.00 | 0.77 | 3.10 | 0.42 | 0.00 |
| *Lumbricus castaneus* | 0.00 | 0.00 | 0.00 | 0.00 | 0.00 | 0.00 | 1.21 | 0.00 | 0.00 |
| *Lumbricus rubellus* | 0.00 | 0.00 | 1.67 | 7.60 | 0.00 | 0.00 | 0.00 | 0.00 | 0.00 |
| *Lumbricus terrestris* | 0.00 | 0.00 | 0.00 | 0.00 | 0.00 | 1.47 | 0.00 | 1.70 | 0.00 |
| *Octolasion* spp. | 1.57 | 0.00 | 0.00 | 0.00 | 0.00 | 0.00 | 0.00 | 0.30 | 0.00 |
| *Octolasion cyaneum* | 4.18 | 0.00 | 0.00 | 0.00 | 0.00 | 0.00 | 0.00 | 1.33 | 0.00 |
| Not determinable | 0.62 | 0.00 | 0.00 | 0.68 | 0.00 | 0.00 | 0.06 | 0.34 | 0.00 |
| Tanylobous species | 0.00 | 0.00 | 2.20 | 8.10 | 0.00 | 2.24 | 4.30 | 2.13 | 0.00 |
| Epilobous species | 10.82 | 0.44 | 0.00 | 0.45 | 0.00 | 1.73 | 5.22 | 8.14 | 0.80 |
| Epigeic adults | 0.00 | 0.44 | 1.67 | 8.00 | 0.00 | 0.00 | 1.21 | 0.00 | 0.13 |
| Endogeic adults | 8.56 | 0.00 | 0.00 | 0.00 | 0.00 | 0.46 | 4.13 | 6.97 | 0.00 |
| Anecic adults | 0.00 | 0.00 | 0.00 | 0.00 | 0.00 | 1.47 | 0.00 | 1.70 | 0.00 |
| Sum of adults | 8.56 | 0.44 | 1.67 | 8.00 | 0.00 | 1.94 | 5.34 | 8.67 | 0.13 |
| Sum of juveniles | 2.26 | 0.00 | 0.53 | 0.54 | 0.00 | 2.03 | 4.18 | 1.59 | 0.67 |
| **Total biomass** | **11.43** | **0.44** | **2.20** | **9.22** | **0.00** | **3.97** | **9.58** | **10.60** | **0.80** |

¹ Not differentiated between the closely related genera *Aporrectodea* and *Allolobophora*

Table S2.7: Number of COI comDNA metabarcoding reads for earthworms at the sampled arable sites

| ASV/Taxon | BIM | GHU | KLK | LIS | NEU | SLT | TRO | WOL |
| --- | --- | --- | --- | --- | --- | --- | --- | --- |
| *Allolobophora chlorotica* | 0 | 0 | 0 | 676 | 9194 | 0 | 0 | 0 |
| *Allolobophora chlorotica* L4 | 0 | 0 | 0 | 2301 | 0 | 0 | 97 | 0 |
| *Allolobophora chlorotica* L5 | 0 | 0 | 0 | 0 | 0 | 0 | 33 | 0 |
| *Aporrectodea* sp. | 0 | 0 | 0 | 0 | 0 | 0 | 13893 | 0 |
| *Aporrectodea caliginosa* | 27987 | 70244 | 42047 | 18777 | 28158 | 47112 | 1423 | 0 |
| *Aporrectodea caliginosa* L1 | 0 | 0 | 0 | 0 | 907 | 0 | 987 | 0 |
| *Aporrectodea caliginosa* L2 | 0 | 1165 | 0 | 2110 | 0 | 0 | 0 | 0 |
| *Aporrectodea caliginosa* L3 | 2679 | 0 | 0 | 445 | 14653 | 0 | 0 | 0 |
| *Aporrectodea icterica* | 0 | 0 | 0 | 7614 | 0 | 0 | 0 | 0 |
| *Aporrectodea longa* | 0 | 0 | 0 | 5830 | 0 | 0 | 6154 | 0 |
| *Aporrectodea rosea* | 0 | 0 | 0 | 0 | 0 | 0 | 259 | 185 |
| *Aporrectodea rosea* L4 | 0 | 3038 | 0 | 10324 | 0 | 0 | 5484 | 22603 |
| *Lumbricus castaneus* | 0 | 0 | 0 | 0 | 0 | 0 | 309 | 0 |
| *Lumbricus rubellus* | 0 | 0 | 0 | 0 | 0 | 12167 | 0 | 0 |
| *Lumbricus terrestris* | 5456 | 12119 | 0 | 0 | 1441 | 0 | 5365 | 19146 |
| *Octolasion cyaneum* | 0 | 0 | 0 | 0 | 0 | 0 | 0 | 13281 |
| *Octolasion tyrtaeum* | 0 | 0 | 0 | 0 | 0 | 0 | 375 | 0 |
| Lumbricidae sp. | 0 | 0 | 0 | 0 | 0 | 1762 | 0 | 0 |
| **No. of ASV/Taxa** | **3** | **4** | **1** | **8** | **5** | **3** | **11** | **4** |

Table S2.8: Number of COI comDNA metabarcoding reads for earthworms at the sampled grassland sites

| ASV/Taxon | ALM | BRU | GAL | KUM | PAU | SHG | ZEC | ZIN |
| --- | --- | --- | --- | --- | --- | --- | --- | --- |
| *Allolobophora chlorotica* | 1209 | 740 | 0 | 0 | 29938 | 8363 | 0 | 0 |
| *Allolobophora chlorotica* L1 | 0 | 0 | 758 | 0 | 0 | 0 | 0 | 0 |
| *Allolobophora chlorotica* L2 | 0 | 20390 | 0 | 0 | 0 | 2586 | 0 | 0 |
| *Allolobophora chlorotica* L3 | 0 | 0 | 0 | 0 | 18204 | 0 | 0 | 0 |
| *Aporrectodea* sp. | 2708 | 201 | 12221 | 14126 | 0 | 584 | 3774 | 0 |
| *Aporrectodea caliginosa* | 7684 | 43324 | 6457 | 32413 | 2965 | 6 | 25130 | 36982 |
| *Aporrectodea caliginosa* L2 | 278 | 0 | 0 | 0 | 0 | 0 | 0 | 0 |
| *Aporrectodea caliginosa* L3 | 1362 | 0 | 3418 | 0 | 0 | 0 | 1400 | 0 |
| *Aporrectodea longa* | 5812 | 17 | 4399 | 0 | 0 | 14583 | 11714 | 0 |
| *Aporrectodea rosea* | 760 | 0 | 4183 | 6640 | 0 | 11419 | 0 | 0 |
| *Aporrectodea rosea* L4 | 6287 | 0 | 6437 | 5237 | 0 | 1903 | 2761 | 0 |
| *Dendrobaena attemsi* | 0 | 0 | 0 | 0 | 0 | 4 | 0 | 0 |
| *Lumbricus castaneus* | 0 | 0 | 27 | 0 | 0 | 0 | 0 | 0 |
| *Lumbricus rubellus* | 304 | 0 | 2459 | 0 | 0 | 0 | 462 | 19247 |
| *Lumbricus rubellus* L2 | 0 | 0 | 0 | 0 | 2554 | 0 | 0 | 0 |
| *Lumbricus terrestris* | 3684 | 0 | 2984 | 13341 | 0 | 14401 | 8846 | 0 |
| *Octolasion* sp. | 0 | 0 | 376 | 0 | 0 | 0 | 0 | 0 |
| *Octolasion cyaneum* | 1203 | 0 | 638 | 17841 | 0 | 0 | 0 | 0 |
| *Octolasion tyrtaeum* | 0 | 0 | 51 | 0 | 0 | 0 | 63 | 0 |
| *Proctodrilus tuberculatus* | 0 | 0 | 0 | 0 | 0 | 18 | 0 | 0 |
| **No. of ASV/Taxa** | **11** | **5** | **13** | **6** | **4** | **10** | **8** | **2** |

Table S2.9: Number of COI comDNA metabarcoding reads for earthworms at the sampled forest sites

| ASV/Taxon | BOV | EUT | FIS | KAN | MER | POS | SBU | SHW | WBU |
| --- | --- | --- | --- | --- | --- | --- | --- | --- | --- |
| *Allolobophora chlorotica* | 0 | 0 | 0 | 0 | 0 | 0 | 3030 | 0 | 0 |
| *Aporrectodea* sp. | 11685 | 0 | 0 | 0 | 0 | 0 | 0 | 0 | 0 |
| *Aporrectodea caliginosa* | 12558 | 0 | 0 | 0 | 0 | 43727 | 14581 | 27299 | 0 |
| *Aporrectodea caliginosa* L2 | 0 | 0 | 0 | 0 | 0 | 0 | 6703 | 0 | 0 |
| *Aporrectodea caliginosa* L3 | 6988 | 0 | 0 | 0 | 0 | 0 | 0 | 0 | 0 |
| *Aporrectodea rosea* | 0 | 0 | 0 | 0 | 0 | 2214 | 0 | 0 | 0 |
| *Aporrectodea rosea* L4 | 8878 | 0 | 0 | 0 | 0 | 0 | 0 | 11848 | 0 |
| *Dendrobaena attemsi* | 0 | 0 | 0 | 0 | 0 | 0 | 0 | 0 | 63404 |
| *Dendrobaena octaedra* | 0 | 0 | 0 | 7278 | 0 | 0 | 0 | 0 | 0 |
| *Dendrodrilus* sp. | 0 | 906 | 0 | 0 | 0 | 0 | 0 | 0 | 0 |
| *Dendrodrilus rubidus* | 0 | 50737 | 0 | 0 | 0 | 0 | 0 | 0 | 0 |
| *Lumbricus castaneus* | 0 | 0 | 0 | 0 | 0 | 0 | 7876 | 0 | 0 |
| *Lumbricus rubellus* | 0 | 0 | 0 | 29854 | 0 | 0 | 3501 | 1611 | 0 |
| *Lumbricus rubellus* L1 | 0 | 0 | 74145 | 0 | 0 | 0 | 0 | 0 | 0 |
| *Lumbricus terrestris* | 0 | 0 | 0 | 0 | 0 | 33447 | 6190 | 2107 | 0 |
| *Octolasion cyaneum* | 7794 | 0 | 0 | 0 | 0 | 0 | 0 | 0 | 0 |
| *Octolasion tyrtaeum* | 0 | 0 | 0 | 0 | 0 | 0 | 0 | 590 | 0 |
| Lumbricidae sp. | 0 | 212 | 0 | 0 | 0 | 0 | 0 | 1244 | 0 |
| **No. of ASV/Taxa** | **5** | **3** | **1** | **2** | **0** | **3** | **6** | **6** | **1** |

Table S2.10: No. of 16S eDNA metabarcoding reads for earthworms at the sampled arable sites

| ASV/Taxon | BIM | KLK | LIS | NEU | SLT | TRO | WOL |
| --- | --- | --- | --- | --- | --- | --- | --- |
| *Allolobophora chlorotica* | 0 | 0 | 4247 | 3882 | 0 | 11 | 0 |
| *Aporrectodea caliginosa* | 15057 | 32212 | 10507 | 14294 | 0 | 5465 | 0 |
| *Aporrectodea icterica* | 0 | 0 | 3541 | 0 | 0 | 0 | 0 |
| *Aporrectodea longa* | 0 | 0 | 45862 | 0 | 1640 | 31067 | 0 |
| *Aporrectodea rosea* | 0 | 0 | 2077 | 5749 | 0 | 15882 | 168 |
| *Dendrobaena octaedra* | 0 | 0 | 0 | 0 | 3090 | 0 | 0 |
| *Lumbricus terrestris* | 0 | 0 | 0 | 36372 | 0 | 9107 | 3636 |
| *Octolasion tyrtaeum* | 0 | 0 | 0 | 0 | 0 | 8741 | 0 |
| **No. of ASV/Taxa** | **1** | **1** | **5** | **4** | **2** | **6** | **2** |

Table S2.11: No. of 16S eDNA metabarcoding reads for earthworms at the sampled grassland sites

| ASV/Taxon | ALM | BRU | GAL | PAU | SHG | ZEC | ZIN |
| --- | --- | --- | --- | --- | --- | --- | --- |
| *Allolobophora chlorotica* | 5081 | 731 | 0 | 44939 | 11819 | 0 | 0 |
| *Aporrectodea caliginosa* | 41227 | 12953 | 1348 | 202 | 7 | 9311 | 0 |
| *Aporrectodea longa* | 10109 | 0 | 0 | 0 | 6833 | 36997 | 10933 |
| *Aporrectodea rosea* | 2565 | 0 | 2284 | 0 | 17323 | 138 | 0 |
| *Dendrobaena octaedra* | 0 | 0 | 0 | 0 | 0 | 0 | 438 |
| *Lumbricus castaneus* | 0 | 0 | 0 | 0 | 144 | 0 | 0 |
| *Lumbricus rubellus* | 3919 | 0 | 0 | 0 | 0 | 37 | 25208 |
| *Lumbricus terrestris* | 0 | 0 | 0 | 0 | 13423 | 7090 | 0 |
| *Octolasion* sp. | 0 | 0 | 23361 | 0 | 0 | 0 | 0 |
| *Octolasion cyaneum* | 3131 | 0 | 0 | 0 | 0 | 0 | 0 |
| *Proctodrilus antipae* | 0 | 0 | 0 | 0 | 1408 | 0 | 0 |
| **No. of ASV/Taxa** | **6** | **2** | **3** | **2** | **7** | **5** | **3** |

Table S2.12: No. of 16S eDNA metabarcoding reads for earthworms at the sampled forest sites

| ASV/Taxon | BOV | EUT | FIS | KAN | MER | SBU | SHW | WBU |
| --- | --- | --- | --- | --- | --- | --- | --- | --- |
| *Aporrectodea caliginosa* | 1401 | 0 | 0 | 0 | 0 | 15729 | 13979 | 0 |
| *Aporrectodea longa* | 0 | 0 | 0 | 0 | 0 | 0 | 0 | 38 |
| *Dendrobaena attemsi* | 0 | 0 | 0 | 0 | 0 | 0 | 0 | 2310 |
| *Dendrobaena octaedra* | 0 | 0 | 43 | 5020 | 0 | 0 | 0 | 0 |
| *Dendrobaena pygmaea* | 0 | 0 | 0 | 1603 | 0 | 0 | 0 | 0 |
| *Dendrodrilus rubidus* | 0 | 1719 | 0 | 0 | 0 | 5610 | 0 | 0 |
| *Lumbricus castaneus* | 0 | 0 | 0 | 0 | 0 | 0 | 189 | 0 |
| *Lumbricus rubellus* | 0 | 0 | 3021 | 439 | 1346 | 440 | 4886 | 0 |
| *Lumbricus terrestris* | 0 | 0 | 0 | 0 | 0 | 5950 | 0 | 0 |
| *Octolasion tyrtaeum* | 0 | 0 | 0 | 0 | 0 | 0 | 163 | 0 |
| Lumbricidae sp. | 0 | 0 | 0 | 0 | 0 | 0 | 0 | 253 |
| **No. of ASV/Taxa** | **1** | **1** | **2** | **3** | **1** | **4** | **4** | **3** |
