# Supplementary material for: Comparison of Morphological and DNA‐Based Identification Methods to Assess Earthworm (Clitellata: Lumbricidae) Diversity at 25 Permanent Soil Monitoring Sites in Germany
Source: Ecol Evol. 2025 Mar 30;15(4):e71155. doi: 10.1002/ece3.71155 (PMC11955243; doi:10.1002/ece3.71155)
Supplement: Supplementary file 3 — Appendices S3. [file ECE3-15-e71155-s004.docx]

**Appendix S3: Comparison of the earthworm species identified by the three identification methods per site**

Figure S3.1: Earthworm species identified jointly and exclusively by morphological identification (morph.), COI comDNA (c-COI) and 16S eDNA (e-16S) metabarcoding in the ALM sampling site

| Species | morph. | comDNA | eDNA |
| --- | --- | --- | --- |
| *Allolobophora chlorotica* |  | * | * |
| *Aporrectodea caliginosa* | * | * | * |
| *Aporrectodea longa* |  | * | * |
| *Aporrectodea rosea* | * | * | * |
| *Lumbricus rubellus* |  | * | * |
| *Lumbricus terrestris* | * | * |  |
| *Octolasion cyaneum* |  | * | * |
| Sum | 3 | 7 | 6 |

Figure S3.2: Earthworm species identified jointly and exclusively by morphological identification (morph.), COI comDNA (c-COI) and 16S eDNA (e-16S) metabarcoding in the BIM sampling site

| Species | morph. | comDNA | eDNA |
| --- | --- | --- | --- |
| *Aporrectodea caliginosa* | * | * | * |
| *Lumbricus terrestris* | * | * |  |
| Sum | 2 | 2 | 1 |

Figure S3.3: Earthworm species identified jointly and exclusively by morphological identification (morph.), COI comDNA (c-COI) and 16S eDNA (e-16S) metabarcoding in the BOV sampling site

| Species | morph. | comDNA | eDNA |
| --- | --- | --- | --- |
| *Aporrectodea caliginosa* | * | * | * |
| *Aporrectodea rosea* | * | * |  |
| *Octolasion cyaneum* | * | * |  |
| Sum | 3 | 3 | 1 |

Figure S3.4: Earthworm species identified jointly and exclusively by morphological identification (morph.), COI comDNA (c-COI) and 16S eDNA (e-16S) metabarcoding in the BRU sampling site

| Species | morph. | comDNA | eDNA |
| --- | --- | --- | --- |
| *Allolobophora chlorotica* | * | * | * |
| *Aporrectodea caliginosa* | * | * | * |
| *Aporrectodea longa* |  | * |  |
| Sum | 2 | 3 | 2 |

Figure S3.5: Earthworm species identified jointly and exclusively by morphological identification (morph.), COI comDNA (c-COI) and 16S eDNA (e-16S) metabarcoding in the EUT sampling site

| Species | morph. | comDNA | eDNA |
| --- | --- | --- | --- |
| *Dendrodrilus rubidus* | * | * | * |
| Sum | 1 | 1 | 1 |

Figure S3.6: Earthworm species identified jointly and exclusively by morphological identification (morph.), COI comDNA (c-COI) and 16S eDNA (e-16S) metabarcoding in the FIS sampling site

| Species | morph. | comDNA | eDNA |
| --- | --- | --- | --- |
| *Dendrobaena octaedra* |  |  | * |
| *Lumbricus rubellus* | * | * | * |
| Sum | 1 | 1 | 2 |

Figure S3.7: Earthworm species identified jointly and exclusively by morphological identification (morph.), COI comDNA (c-COI) and 16S eDNA (e-16S) metabarcoding in the GAL sampling site

| Species | morph. | comDNA | eDNA |
| --- | --- | --- | --- |
| *Allolobophora chlorotica* | * | * |  |
| *Aporrectodea caliginosa* | * | * | * |
| *Aporrectodea longa* | * | * |  |
| *Aporrectodea rosea* | * | * | * |
| *Lumbricus castaneus* |  | * |  |
| *Lumbricus rubellus* | * | * |  |
| *Lumbricus terrestris* | * | * |  |
| *Octolasion cyaneum* | * | * |  |
| *Octolasion tyrtaeum* | * | * |  |
| Sum | 8 | 9 | 2 |

Figure S3.8: Earthworm species identified jointly and exclusively by morphological identification (morph.) and COI comDNA (c-COI) metabarcoding in the GHU sampling site

| Species | morph. | comDNA |
| --- | --- | --- |
| *Aporrectodea caliginosa* | * | * |
| *Aporrectodea rosea* | * | * |
| *Lumbricus terrestris* | * | * |
| Sum | 3 | 3 |

Figure S3.9: Earthworm species identified jointly and exclusively by morphological identification (morph.), COI comDNA (c-COI) and 16S eDNA (e-16S) metabarcoding in the KAN sampling site

| Species | morph. | comDNA | eDNA |
| --- | --- | --- | --- |
| *Dendrobaena octaedra* | * | * | * |
| *Dendrobaena pygmaea* |  |  | * |
| *Dendrodrilus rubidus* |  |  | * |
| *Lumbricus rubellus* | * | * | * |
| Sum | 2 | 2 | 4 |

Figure S3.10: Earthworm species identified jointly and exclusively by morphological identification (morph.), COI comDNA (c-COI) and 16S eDNA (e-16S) metabarcoding in the KLK sampling site

| Species | morph. | comDNA | eDNA |
| --- | --- | --- | --- |
| *Aporrectodea caliginosa* | * | * | * |
| Sum | 1 | 1 | 1 |

Figure S3.11: Earthworm species identified jointly and exclusively by morphological identification (morph.) and COI comDNA (c-COI) metabarcoding in the KUM sampling site

| Species | morph. | comDNA |
| --- | --- | --- |
| *Aporrectodea caliginosa* | * | * |
| *Aporrectodea rosea* | * | * |
| *Lumbricus terrestris* | * | * |
| *Octolasion cyaneum* | * | * |
| Sum | 4 | 4 |

Figure S3.12: Earthworm species identified jointly and exclusively by morphological identification (morph.), COI comDNA (c-COI) and 16S eDNA (e-16S) metabarcoding in the LIS sampling site

| Species | morph. | comDNA | eDNA |
| --- | --- | --- | --- |
| *Allolobophora chlorotica* | * | * | * |
| *Aporrectodea caliginosa* | * | * | * |
| *Aporrectodea icterica* |  | * | * |
| *Aporrectodea longa* | * | * | * |
| *Aporrectodea rosea* | * | * | * |
| Sum | 4 | 5 | 5 |

Figure S3.13: Earthworm species identified jointly and exclusively by morphological identification (morph.), COI comDNA and 16S eDNA metabarcoding in the MER sampling site

| Species | morph. | comDNA | eDNA |
| --- | --- | --- | --- |
| *Lumbricus rubellus* |  |  | * |
| Sum | 0 | 0 | 1 |

Figure S3.14: Earthworm species identified jointly and exclusively by morphological identification (morph.), COI comDNA and 16S eDNA metabarcoding in the NEU sampling site

| Species | morph. | comDNA | eDNA |
| --- | --- | --- | --- |
| *Allolobophora chlorotica* | * | * | * |
| *Aporrectodea caliginosa* | * | * | * |
| *Aporrectodea rosea* |  |  | * |
| *Lumbricus terrestris* | * | * | * |
| Sum | 3 | 3 | 4 |

Figure S3.15: Earthworm species identified jointly and exclusively by morphological identification (morph.), COI comDNA (c-COI) and 16S eDNA (e-16S) metabarcoding in the PAU sampling site

| Species | morph. | comDNA | eDNA |
| --- | --- | --- | --- |
| *Allolobophora chlorotica* | * | * | * |
| *Aporrectodea caliginosa* |  | * | * |
| *Aporrectodea longa* | * |  |  |
| *Lumbricus rubellus* | * | * |  |
| Sum | 3 | 3 | 2 |

Figure S3.16: Earthworm species identified jointly and exclusively by morphological identification (morph.) and COI comDNA (c-COI) metabarcoding in the POS sampling site

| Species | morph. | comDNA |
| --- | --- | --- |
| *Aporrectodea caliginosa* | * | * |
| *Aporrectodea rosea* |  | * |
| *Lumbricus terrestris* | * | * |
| Sum | 2 | 3 |

Figure S3.17: Earthworm species identified jointly and exclusively by morphological identification (morph.), COI comDNA (c-COI) and 16S eDNA (e-16S) metabarcoding in the SBU sampling site

| Species | morph. | comDNA | eDNA |
| --- | --- | --- | --- |
| *Allolobophora chlorotica* |  | * |  |
| *Aporrectodea caliginosa* | * | * | * |
| *Dendrodrilus rubidus* |  |  | * |
| *Lumbricus castaneus* | * | * |  |
| *Lumbricus rubellus* |  | * | * |
| *Lumbricus terrestris* |  | * | * |
| Sum | 2 | 5 | 4 |

Figure S3.18: Earthworm species identified jointly and exclusively by morphological identification (morph.), COI comDNA (c-COI) and 16S eDNA (e-16S) metabarcoding in the SHG sampling site

| Species | morph. | comDNA | eDNA |
| --- | --- | --- | --- |
| *Allolobophora chlorotica* | * | * | * |
| *Aporrectodea caliginosa* |  | * | * |
| *Aporrectodea longa* | * | * | * |
| *Aporrectodea rosea* | * | * | * |
| *Dendrobaena attemsi* |  | * |  |
| *Lumbricus castaneus* |  |  | * |
| *Lumbricus terrestris* | * | * | * |
| *Proctodrilus antipae* | * |  | * |
| *Proctodrilus tuberculatus* |  | * |  |
| Sum | 5 | 7 | 7 |

Figure S3.19: Earthworm species identified jointly and exclusively by morphological identification (morph.), COI comDNA (c-COI) and 16S eDNA (e-16S) metabarcoding in the SHW sampling site

| Species | morph. | comDNA | eDNA |
| --- | --- | --- | --- |
| *Aporrectodea caliginosa* | * | * | * |
| *Aporrectodea rosea* | * | * |  |
| *Lumbricus castaneus* |  |  | * |
| *Lumbricus rubellus* |  | * | * |
| *Lumbricus terrestris* | * | * |  |
| *Octolasion cyaneum* | * | * |  |
| *Octolasion tyrtaeum* |  | * | * |
| Sum | 4 | 6 | 4 |

Figure S3.20: Earthworm species identified jointly and exclusively by morphological identification (morph.), COI comDNA (c-COI) and 16S eDNA (e-16S) metabarcoding in the SLT sampling site

| Species | morph. | comDNA | eDNA |
| --- | --- | --- | --- |
| *Aporrectodea caliginosa* | * | * | * |
| *Dendrobaena octaedra* |  |  | * |
| *Lumbricus rubellus* | * | * |  |
| Sum | 2 | 2 | 2 |

Figure S3.21: Earthworm species identified jointly and exclusively by morphological identification (morph.), COI comDNA (c-COI) and 16S eDNA (e-16S) metabarcoding in the TRO sampling site

| Species | morph. | comDNA | eDNA |
| --- | --- | --- | --- |
| *Allolobophora chlorotica* | * | * | * |
| *Aporrectodea caliginosa* | * | * | * |
| *Aporrectodea longa* | * | * | * |
| *Aporrectodea rosea* | * | * | * |
| *Lumbricus castaneus* |  | * |  |
| *Lumbricus terrestris* | * | * | * |
| *Octolasion tyrtaeum* | * | * |  |
| Sum | 6 | 7 | 5 |

Figure S3.22: Earthworm species identified jointly and exclusively by morphological identification (morph.), COI comDNA (c-COI) and 16S eDNA (e-16S) metabarcoding in the WBU sampling site

| Species | morph. | comDNA | eDNA |
| --- | --- | --- | --- |
| *Aporrectodea longa* |  |  | * |
| *Dendrobaena attemsi* | * | * | * |
| Sum | 1 | 1 | 2 |

Figure S3.23: Earthworm species identified jointly and exclusively by morphological identification (morph.), COI comDNA (c-COI) and 16S eDNA (e-16S) metabarcoding in the WOL sampling site

| Species | morph. | comDNA | eDNA |
| --- | --- | --- | --- |
| *Aporrectodea rosea* | * | * | * |
| *Lumbricus terrestris* | * | * | * |
| *Octolasion cyaneum* |  | * |  |
| Sum | 2 | 3 | 2 |

Figure S3.24: Earthworm species identified jointly and exclusively by morphological identification (morph.), COI comDNA (c-COI) and 16S eDNA (e-16S) metabarcoding in the ZEC sampling site

| Species | morph. | comDNA | eDNA |
| --- | --- | --- | --- |
| *Aporrectodea caliginosa* | * | * | * |
| *Aporrectodea longa* | * | * | * |
| *Aporrectodea rosea* | * | * | * |
| *Lumbricus rubellus* |  | * | * |
| *Lumbricus terrestris* | * | * | * |
| *Octolasion tyrtaeum* |  | * |  |
| Sum | 4 | 6 | 5 |

Figure S3.25: Earthworm species identified jointly and exclusively by morphological identification (morph.), COI comDNA (c-COI) and 16S eDNA (e-16S) metabarcoding in the ZIN sampling site

| Species | morph. | comDNA | eDNA |
| --- | --- | --- | --- |
| *Aporrectodea caliginosa* | * | * | * |
| *Dendrobaena octaedra* |  |  | * |
| *Lumbricus rubellus* | * | * | * |
| Sum | 2 | 2 | 3 |
