# Supplementary material for: Comparison of Morphological and DNA‐Based Identification Methods to Assess Earthworm (Clitellata: Lumbricidae) Diversity at 25 Permanent Soil Monitoring Sites in Germany
Source: Ecol Evol. 2025 Mar 30;15(4):e71155. doi: 10.1002/ece3.71155 (PMC11955243; doi:10.1002/ece3.71155)
Supplement: Supplementary file 4 — Appendices S4. [file ECE3-15-e71155-s003.docx]

**Appendix S4: Additional correlation diagrams between the relative abundance and biomass of species and the relative number of eDNA metabarcoding reads**

Figure S4.1: Correlation between the relative abundance of earthworm species and the relative number of eDNA metabarcoding reads (see Table 1 for site abbreviations)

Figure S4.2: Correlation between the relative biomass of earthworm species and the relative number of eDNA metabarcoding reads (see Table 1 for site abbreviations)

Figure S4.3: Correlation between the relative abundance and biomass of *Allolobophora chlorotica* and the relative number of eDNA metabarcoding reads (see Table 1 for site abbreviations)

Figure S4.4: Correlation between the relative abundance and biomass of *Aporrectodea caliginosa* and the relative number of eDNA metabarcoding reads (see Table 1 for site abbreviations)

Figure S4.5: Correlation between the relative abundance and biomass of *Aporrectodea rosea* and the relative number of eDNA metabarcoding reads (see Table 1 for site abbreviations)

Figure S4.6: Correlation between the relative abundance and biomass of *Aporrectodea longa* and the relative number of eDNA metabarcoding reads (see Table 1 for site abbreviations)

Figure S4.7: Correlation between the relative abundance and biomass of *Lumbricus terrestris* and the relative number of eDNA metabarcoding reads (see Table 1 for site abbreviations)
